# A computational framework to study the etiology of grandiose narcissism

**DOI:** 10.1038/s41598-025-90109-w

**Published:** 2025-02-18

**Authors:** Deborah M. Löschner, Martin Schoemann, Emanuel Jauk, Lena Herchenhahn, Sarah Schwöbel, Philipp Kanske, Stefan Scherbaum

**Affiliations:** 1 Institute of Work, Organisational and Social Psychology, Technische Universität Dresden, 01069 Dresden, Germany; 2 Institute of General Psychology, Biopsychology and Methods of Psychology, Technische Universität Dresden, 01069 Dresden, Germany; 3 Department of Medical Psychology, Psychosomatics, and Psychotherapy, Medical University of Graz, 8036 Graz, Austria; 4 Institute of Psychology, Christian-Albrechts-Universität Zu Kiel, 24118 Kiel, Germany; 5 Institute of Clinical Psychology and Psychotherapy, Technische Universität Dresden, 01187 Dresden, Germany

**Keywords:** Etiology, Narcissism, Agent-based modeling, Computational psychology, Personality, Human behaviour, Risk factors

## Abstract

Grandiose narcissism is characterized by ambivalent interaction behavior (i.e., grandiose self-presentation and rivalrous devaluation of others) and strong oscillations in self-esteem over time. In the light of emotional and social problems associated with these self-esteem regulation patterns and the increasing prevalence of narcissistic tendencies, causal and formalized models for prevention and intervention are needed. Here, we present a computational model of narcissistic self-esteem regulation implementing established, verbal theories of narcissism to identify key etiological and disorder-maintaining mechanisms. Across four studies, we show that parental praise and overvaluation lead to typical grandiose-narcissistic behavioral patterns (i.e., entitled self-presentation and rivalry) and strong self-esteem oscillations. Underlying these phenomena, we identify two maintaining mechanisms that offer targets for intervention and empirical falsification: tolerance development, characterized by an ever-increasing desire for social recognition, and a vicious cycle of rivalry, characterized by the frequent use of other-devaluing behavior and massive drops in self-esteem.

## Introduction

Narcissism is a personality dimension characterized by self-idealizing and other-devaluating behavior, as well as egocentric and antagonistic behavior. Especially grandiose narcissism has garnered recent public and scientific attention through the debate about narcissism in CEOs and political leaders, which might make them attractive yet bears great destructive potential^1–3^. This apparent contradiction of charming and extraverted as well as combative and defensive social behavior reflects the crucial agentic and antagonistic characteristics of grandiose narcissism (following multi-dimensional approaches^4,5^). We consider grandiose narcissism as a personality dimension, with more extreme manifestations being associated with expressions of narcissistic vulnerability such as fragile self-esteem and psychological distress^5–7^. Throughout the paper, we refer to this conceptualization of grandiose narcissism when using the term narcissism. While there is extensive research and theory building on how and why these dynamics of grandiose narcissism develops, causal factors cannot be studied experimentally in humans for ethical and economic reasons. To bridge this gap, we can use computational models, for example agent-based models, to simulate interactions between different individuals (e.g., parents and children) and their social consequences under extreme conditions. We propose that using such computational modeling allows us to simulate the causal-developmental conditions of narcissism.

Two lines of etiological theories draw on narcissism-imprinting parenting styles to explain the genesis of narcissism^8^. One strand focuses on permissive over-parenting: An excessive admiration of a child and the conveyance that it is special and better than others^9^ leads to a self-conception as being grandiose and entitled. The other strand focuses on indifferent under-parenting: A perceived lack of parental warmth is compensated by the child with an inflated self-view and externalizing behavior (e.g., seeking attention and validation^10–13^). Regarding parental feedback for children’s behavior, both parenting styles lead to similar consequences: The child lacks the ability to accurately evaluate the effectiveness of their own actions and is unable to experience appropriate levels of frustration^14^ as well as to form a realistic perception of themselves^13,15^. The empirical evidence for either strand, however, is rather heterogeneous^15–22^ and inherently stems from retrospective, epidemiological, and longitudinal studies^8^: Retrospective studies link self-assessment measures of individuals’ retrospective perceptions of their parents’ characteristics with narcissism-related measures (e.g., association between parental maltreatment and narcissism^21^). Epidemiological studies assess narcissism-related scores in large samples across multiple groups and examine the relationship between distinct group or cultural characteristics and narcissism scores (e.g., comparison of narcissism-expression between East and West Germany^22^). Longitudinal studies assess static measures of narcissism and parental characteristics over multiple measurement points, observing changes over time (e.g., assessing parenting styles of subjects parents at age 3 and their narcissism scores at age 23^18^). Such methodological approaches fail to account for the mechanisms and temporal dynamics that underpin associations between static constructs. Hence, there is a lack of direct causal evidence for an assumed relationship.

To address this lack of causal evidence, modeling and simulation studies have proven useful in other areas. During the COVID-19 pandemic, modeling enabled the simulation of how specific characteristics, such as the virus’ reproduction number or implementation of lockdowns, causally impact the behavior of a system, such as the mortality rate^23^. More specifically, agent-based modeling was employed to elucidate how behavioral dynamics at a micro level, encompassing intra-individual factors (e.g., willingness to be vaccinated), inter-individual factors (e.g., staying at home versus meeting friends) and environmental factors (e.g., lockdowns) causally impact macro level processes, such as infection rates^23^. Potential influencing factors (e.g., vaccination rate) are in temporal sequence linked with relevant outcomes (e.g., mortality rate), thereby establishing causality. In this way, we can depict hidden mechanisms behind observable states (e.g., the precise influence of an intervention on the mortality rate). As a result, the “black box” of mechanisms underlying observable behavior may be explored better.

In this article, we unpack the “black box” of etiological and maintaining dynamics of narcissism and identify the hidden causal mechanisms behind patterns of narcissistic behavior observed in the real world. Using agent-based modeling, we test how etiological macro-level assumptions shape behavioral dynamics on a micro level and draw inferences from empirically demonstrated characteristics to underlying processes. Thereby, we build a link between etiological assumptions of narcissism and measurable phenotypic states that psychology summarizes under the term “narcissistic”. The core components for such a model are similar to the models that were used during the COVID-19 pandemic: environmental conditions (i.e., parenting styles as described above) as well as intra- and inter-individual processes (e.g., individual characteristics like an inflated self-view or specific forms of interaction behavior). These three components are inherent to agent-based models: Agents each have intra-individual features implemented which define their behavior and reactions to the behavior of others. In a defined setting, they are enabled to interact with one another and can be influenced by environmental phenomena.

### The etiology of narcissism as an agent-based model

Environmental conditions are reflected in different parenting styles leading to different learning experiences. According to the different etiological theories, an inflated, unrealistic self-view in narcissism may evolve from diverging learning experiences. Parents’ excessive overvaluation or detached indifference shape the child’s self-view and expectation of others’ behavior^9,17^. In an overvaluing environment, the self-view corresponds to the learned expectations from the environment ("I am simply the best."). In a devaluing environment, this self-view serves to compensate for the experienced devaluation and thus to protect the self ("The others just don’t know how good I am.”^10^). Thus, the self-view is the temporally stable expectation regarding one’s own self-esteem. If the self-esteem does not correspond to this actual expected value, the resulting dissonance mobilizes behavior for self-esteem regulation^24,25^. Two crucial characteristics show the special relevance of this interplay of self-view, self-esteem, and self-esteem regulation in narcissism: strong self-esteem oscillations over time^26,27^ and frequent use of inter-individual self-esteem regulation strategies^28,29^.

Baumeister and Vohs^30^ describe narcissism as an "addiction to self-esteem" and thus also explain the characteristic strong oscillations in self-esteem. Self-esteem depends decisively on external validation: if the narcissistic person obtains external approval, their self-esteem increases. Narcissists can never get enough affirmation, the environment cannot permanently meet this need for external validation. Self-esteem enhancing experiences are therefore repeatedly interrupted by frustrating experiences that lower self-esteem. The result is a strongly oscillating self-esteem. This builds a bridge between empirical evidence for oscillating self-esteem^26,27,31,32^ and an increased sense of entitlement and need for admiration in narcissism^5,33,34^. A change in self-esteem is therefore associated with a change in regulation behavior and vice versa^24,35^.

Regulation of self-esteem can take place internally or externally^24^. Internal, intra-individual regulation of self-esteem includes strategies that a person can perform independently of others, such as reminding oneself of positive experiences or activating positive beliefs/schemas (which may include internalized interpersonal experiences^32^; or grandiose future-oriented fantasies^36^). External, inter-individual regulation includes strategies for which an interaction partner is needed. Two common narcissistic interaction styles to regulate self-esteem are agentic (i.e., desire for admiration, self-presentation) and antagonistic (i.e., rivalry, self-protection) strategies^28,29^. Hence, the apparent contradiction between agentic and antagonistic strategies characterizes grandiose narcissism as ambivalent behavioral dynamics (cf.^35^) with possible shifts between grandiose and vulnerable, self-convinced and fragile, entitled and ashamed, charming and aggressive states^5,28,29,37^. This ambivalence is also apparent in the varying functionality for self-enhancement of both strategies, depending on the strategy itself and environmental factors like the personal status of a person in a group^32^. Admiration as an agentic strategy and the pursuit of uniqueness as well as the indulgence in grandiose fantasies can be socially acceptable and increase self-esteem. Rivalry as an antagonistic strategy and an aggressive devaluation of others when facing a threat to the self-view can result in social conflict and subsequently decrease self-esteem. Self-esteem appears stable at high levels over time with primarily presented admiration behaviors, whereas rivalry appears associated with stronger oscillations at lower levels^26,28,38^.

Bringing these three components (i.e., environmental conditions, internal and external processes) together, we developed a theory-driven, agent-based model to explore mechanisms of self-esteem regulation in grandiose narcissism. Individual agents with specific characteristics (e.g., state self-esteem, predisposition to use admiration for self-esteem regulation) move within a predefined space in which they can encounter and interact with other agents, exhibiting inter-individual self-esteem regulating behavior. We outline the structure of the model and perform four simulation studies, which investigate how learned self-esteem regulation leads to narcissistic behavior patterns, that is admiration and rivalry (Study 1–3), and whether the model can replicate real world data from an ecological momentary assessment study (Study 4). Hence, in this study we pursue an exploratory approach intended to contribute to the theoretical understanding of dynamical mechanisms underlying observable states. As outlined before, our approach is based on the central assumption of the association between self-esteem oscillations and inter-individual self-esteem regulation. Based on this assumption, we follow a pattern-oriented modeling approach to investigate related behavioral dynamics^39^.

## Methods

### Model structure

**Fig. 1:**
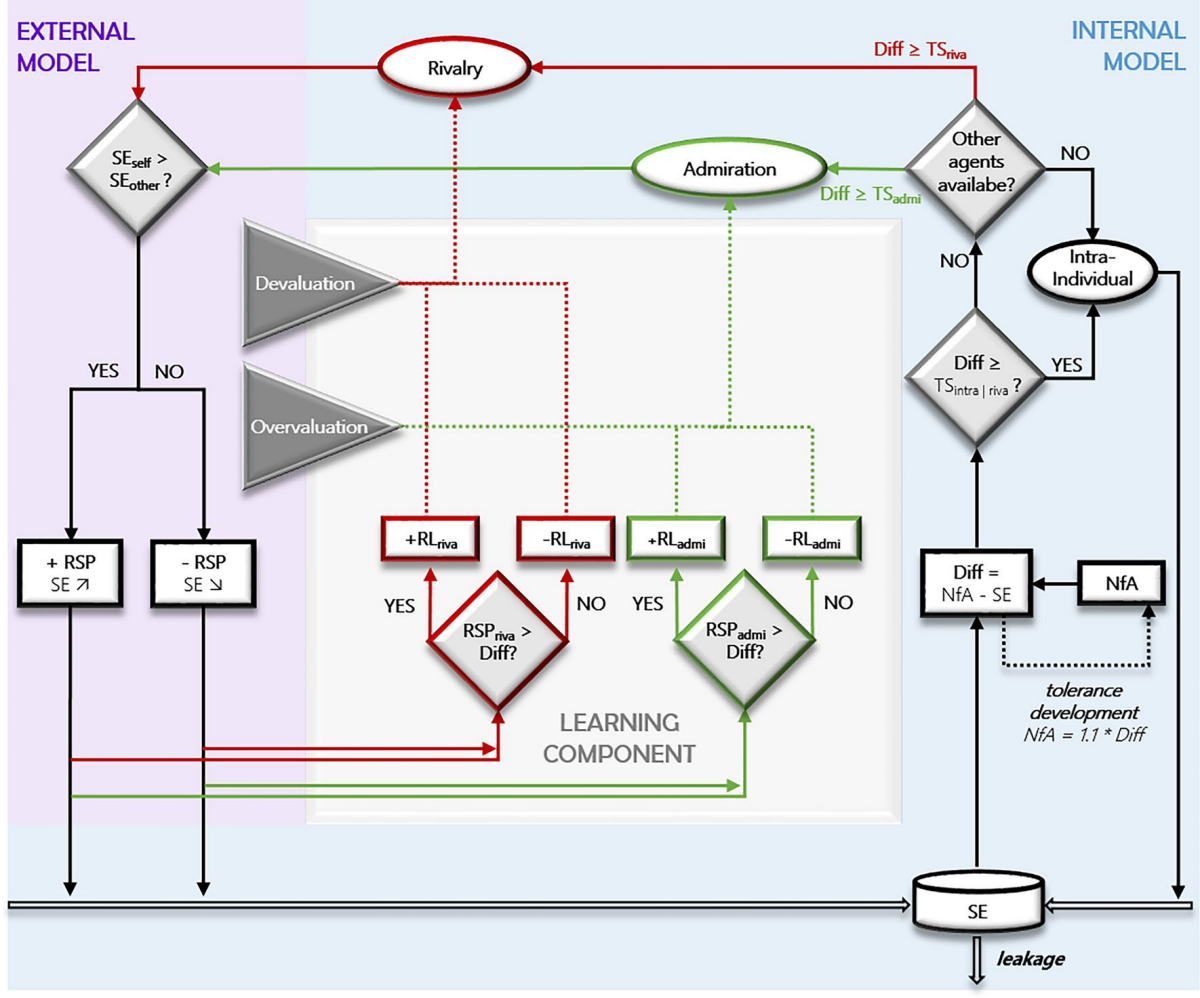
Each agent’s self-esteem (SE) decreases over time (leakage) and increases through regulation behavior (intra-individual, admiration, rivalry). Agents detect their self-esteem at each timestep and compare it with the need for admiration (NfA), conceptualized as the expectancy value of the self-esteem. The difference (Diff) between both of these values is conceptualized as the need for self-esteem regulation and initializes two mechanisms: self-esteem regulation and tolerance development. Due to tolerance development, the need for admiration adapts to self-esteem values in the long run, i.e. it increases with higher self-esteem and decreases with lower self-esteem. If the difference between self-esteem and feed for admiration exceeds certain threshold values (TS_intra_, TS_admi_, TS_riva_), self-esteem regulation behavior is triggered. These behaviors are hierarchically structured. Intra-individual regulation has the lowest threshold (TS_intra_) and affects self-esteem directly; self-esteem increases. If the difference between self-esteem and need for admiration exceeds the thresholds for inter-individual behavior (TS_admi_ and TS_riva_), the agent checks whether possible interaction partners, i.e. other agents, are nearby and thus available. If this is not the case, intra-individual self-esteem regulation is used. If another agent is available (if there are several, one is selected randomly), it is checked whether the difference between self-esteem and need for admiration is greater than the threshold for admiration (TS_admi_) or rivalry (TS_riva_). The behavior with the highest exceeded threshold value is initialized. Admiration is associated with a higher probability of receiving positive feedback, while rivalry is associated with a higher probability of receiving negative feedback. Depending on whose interaction partner self-esteem is higher (SE_self_ vs. SE_other_), the reaction is positive (+ RSP; self-esteem increases) or negative (− RSP; self-esteem decreases). See Supplementary Text C.1 for the exact equations for computing the behavior-dependent response. When the learning component is active, the agent derives learning experiences based on the reaction to admiration or rivalry. If the reaction (RSP_riva_/RSP_admi_; i.e. the effect on self-esteem) is greater than the difference between self-esteem and need for admiration (i.e. large enough to satisfy the need for regulation), this behavior is positively reinforced (+ RL_riva_/ + RL_admi_) and the threshold for initiating the behavior decreases, making the use of the behavior more likely. If the reaction is not greater than the difference between self-esteem and need for admiration, i.e. a need for regulation remains, the behavior is reinforced negatively (− RL_riva_, − RL_admi_) and the threshold for initiating the behavior increases, making it less likely to be used. In addition to these reinforcement learning (RL)-based experiences, there are two external influences that affect the thresholds of inter-individual regulation behavior. Overvaluation describes the non-contingent appreciation of an agent and lowers the admiration threshold, i.e. admiration occurs more frequently. Devaluation describes the non-contingent depreciation of the agent and lowers the rivalry threshold, i.e. rivalry occurs more frequently. Agents move randomly to another cell of a 10 × 10 grid at each timestep.

Our model structure is based on six theoretical assumptions to depict grandiose narcissistic self-esteem regulation dynamics: the Self-Discrepancy Theory^40,41^, the Hierometer Theory^42^, the assumption of leaky self-esteem^24,30^, the Narcissism Admiration and Rivalry theory^28,29^, the etiological theories of narcissism-imprinting parenting styles^9–13,16,^, and assumptions on basic Reinforcement Learning mechanisms^43–45^. We formalized these six assumptions forming a structure of different intertwined control circuits regulating self-esteem (see Fig. 1) of which we present each part in detail in the following.

#### Self-esteem regulation model

The Self-Esteem Regulation (SER) model builds on the evidence of strong oscillating self-esteem in grandiose narcissism^27,32^ and its association to inter-individual regulation behavior^26,33^. It links these two core components to the behavior-mobilizing difference between need for admiration and self-esteem, the responses of other agents to the inter-individual regulation behavior (admiration or rivalry) and the self-esteem leakage. These theoretical assumptions lead to the SER model depicted in Fig. 1. The comprehensive model estimation of self-esteem in each new iteration is depicted in the equation

$${SE}_{t+1}= {SE}_{t}+{RSP}_{t({A}_{t},{S}_{t})}-{L}_{t({SE}_{t})}$$

where (${SE}_{t+1}$) represents the self-esteem in the next iteration, (${SE}_{t}$) the current self-esteem, (${RSP}_{t}$) the response of other agents to self-esteem regulation behavior, which depends on the employed self-esteem regulation behavior (${A}_{t}$ for action), and the status (${S}_{t}$) of the agent in a social hierarchy, and the relative self-esteem leakage (${L}_{t}$) in each iteration. The mathematical formalization of single model components and dynamics is described in Supplementary Text C.1.

#### Learning component (LSER model)

The learning component builds on assumptions on the learning of behavioral predispositions for self-esteem regulation behavior through the adjustment of behavior-initiation thresholds for admiration and rivalry to environmental feedback. We focus on two learning processes, namely non-contingent narcissism-imprinting parenting styles (over- vs. devaluation) as external influences (non-contingent learning) and contingent basic reinforcement learning mechanisms controlling for the responses to the shown self-esteem regulation behavior (contingent learning). These theoretical assumptions lead to the gray shaded extension (learning component) of the SER model depicted in Fig. 1. The comprehensive estimation of the learning experiences in each iteration is formalized in the equation

$${TS}_{A | t+1}={TS}_{A | t}-{NCL}_{t}-{CL}_{t}$$

where $({TS}_{A})$ represents the threshold to activate ($A$) inter-individual regulation behavior (admiration or rivalry), ${(NCL}_{t})$ the non-contingent learning experience and ${(CL}_{t})$ the contingent learning experience. The current threshold and both learning experiences are labeled with “t” and form the threshold at time t + 1 (${TS}_{t+1}$). The exact formalization and theoretical assumptions behind the single equation components can be found in Supplementary Text C.1.

#### Model calibration and validation

To calibrate and validate the model, we followed standard procedures^46–48^. We defined self-esteem oscillations as target pattern. Based on the calibrated default values of the individual model parameters, we conducted tests with extreme values deviating from these defaults to determine the model’s sensitivity to changes in single parameters (see Supplementary Text C.2 and Supplementary Table S1 and S2).

### Simulation studies

#### Study 1: inter-individual self-esteem regulation and self-esteem

In our first simulation study, we tested the effects of different admiration and rivalry thresholds on self-esteem and self-esteem regulation behavior. We ran the basic SER model (with a deactivated learning component) with systematically varying thresholds to activate the self-esteem regulation behaviors admiration and rivalry, and observed variations in self-esteem as well as in regulation behavior frequencies. We created a baseline condition by calibrating the thresholds to default values under which the model showed only minor self-esteem oscillations and no inter-individual regulation behaviors. In the other conditions, we tested three thresholds for activating admiration and rivalry with values of 0.0055 (low), 0.025 (medium), and 0.05 (high), where lower values represent lower thresholds. These threshold values were selected based on the calibration results of the model. We identified three distinct value ranges: Values for which the model shows stable, meaningful behavior and is not sensitive to changes (i.e., threshold values equal to default and higher values), values for which the model shows meaningful, interpretable results behavior and is sensitive to changes (i.e., values between 0.0055 and the default value), and values for which the model no longer shows meaningful, interpretable results (i.e., values smaller than 0.0055). Details regarding the calibration process and sensitivity analysis can be found in Supplementary Text C.2.

Except for the tested threshold value, all other values were set to the default values used in the baseline condition. For each test, we ran the model 50 times over 200 timesteps with a burn-in phase of 50 timesteps and averaged the results of the “narcissistic” agent across all runs. The averaged results can be found in Supplementary Table S3.

#### Study 2: simulation experiments in the learning environment

This study aimed to examine the influence of different learning environments on the development of narcissism-related traits such as frequent use of admiration and rivalry. We tested the influence of two opposing theories: the overvaluation and praise theory^9,16^, positing that narcissistic behavior arises from excessive admiration, and the devaluation and indifference theory^10–13^, positing that narcissistic behavior is fostered by cool neglect and devaluation. Hence, we ran the LSER model (with an activated learning component) in three different environments, and observed the thresholds to activate the self-esteem regulation behaviors admiration and rivalry as well as the self-esteem course over time. We created three environments: (a) an overvaluing and praising environment with non-contingent positive and contingent positive feedback, (b) a devaluing and indifferent environment with non-contingent devaluation and contingent indifferent feedback, and (c) a control environment with contingent feedback according to the used regulation behavior, that is mostly positive for admiration, mostly negative for rivalry (see Supplementary Table S4 for a detailed description of the parameters of all three conditions). The control environment corresponds to the default feedback in the SER model which was used in Study 1. In each learning environment, feedback on displayed behavior and the likelihood of non-contingent feedback were experimentally manipulated. The learning model was run 50 times over 1000 timesteps with one learning agent in each condition. As dependent variable, we analyzed the averaged threshold values for the activation of admiration or rivalry across all runs of the narcissistic agent. In addition to the absolute values, we also examined the variance between the runs (i.e., among all “narcissistic” agents). In order to ensure learning only within the calibrated model values, the lowest calibrated threshold value for the thresholds for admiration and rivalry were set as the lower limits. A detailed overview of all the results can be found in Supplementary Table S5.

#### Study 3: simulation experiments with learned agents

Following assumptions of narcissistic self-esteem regulation as a rigid behavioral pattern that cannot be easily adjusted to changing environmental characteristics^9,49^, typically “narcissistic” self-esteem regulation patterns should particularly manifest when the current environment no longer matches the learning environment. Based on the results of Study 2, we ran the SER model with the average learned thresholds from Study 2 (see Supporting Table S5) and exposed all agents to the SER environment with deactivated learning component. We ran the model for 1000 timesteps and observed individual variations in the self-esteem as well as regulation behavior frequencies. Particularly, we focused on the analysis of typical patterns of narcissistic self-esteem regulation.

#### Study 4: replicating real world data with simulated data

In order to externally validate that the model behavior aligns with lifelike phenomena regarding self-esteem regulation, we validated it externally using real world data. Therefore, data from an ecological momentary assessment (EMA) conducted in Dresden in 2018–2019 was analyzed (for a detailed sample and procedure description see^4^; data available at https://osf.io/jdwav/). Over 1200 participants completed an online-screening, where their scores in grandiose and vulnerable narcissism were measured with the Narcissistic Personality Inventory (NPI^50^) and the Hypersensitive Narcissism Scale (HSNS^51^). Following a continuous orthogonal design in order to maximize variance between grandiose and vulnerable narcissism scores, 167 individuals (84 women, 83 men) with a mean age of 25.72 years (SD_age_ = 6.63, range_age_ = 18–57) from this screening pool were invited to participate in the EMA study. They were categorized into four different groups based on their scores in vulnerable (HSNS) and grandiose (NPI) narcissism (low vs. high). This categorization resulted in the following four groups: (1) low grandiose, low vulnerable (LG,LV), (2) high grandiose, low vulnerable (HG,LV), (3) low grandiose, high vulnerable (LG, HV), (4) high grandiose, high vulnerable (HG,HV) narcissism. Scales of the Five Factor Narcissism Inventory (FFNI^52^) were assessed on a trait level as well, while self-esteem was measured on a state level six times a day with a three item scale asking for the current self-assessment and perception using a custom-developed smartphone application app^4^. All four narcissism groups differed in terms of the FFNI values in agency and antagonism as well as the self-esteem mean and variance (see Supplementary Table S6).

To parallel the real world data, we simulated data according to the four groups to validate the models capability to depict varying self-esteem courses over time on a group level. There is strong evidence for the assumption that narcissistic agency is associated with admiration as a self-regulation strategy, while antagonism is related to rivalry^53,54^. Based on this evidence, we synchronized the FFNI values for agency and antagonism of the EMA data with the thresholds for admiration (TS_admi_) and rivalry (TS_riva_) in the model to simulate the four different groups (see Supplementary Text C.3 and Supplementary Table S6 for the exact procedure and values). Please note that the FFNI values are traits (i.e., the higher the value, the higher the trait expression) which are conceptualized in the model as thresholds (i.e., the lower the values, the higher the trait expression).

Hence, we compared the FFNI variables with the following parameters of the simulated data: the *admiration threshold* which represents the tendency for extraverted self-presentation and depicts a model heuristic of FFNI *agency*, the *rivalry threshold* which represents the tendency for the devaluation of others and depicts a model heuristic of the FFNI *antagonism*, *self-esteem median* which represents the median of the model parameter self-esteem for each agent over time and equals the EMA self-esteem median, and *self-esteem variance* which represents the variance of the model parameter self-esteem for each agent over time and equals the self-esteem variance.

The self-esteem-related measures match in both datasets. For the trait variables, the labels used in the EMA data are adopted. Therefore, in the following *agency* represents FFNI *agency* and the *admiration threshold*, and *antagonism* represents FFNI *antagonism* and the *rivalry threshold*.

We examined both the thresholds for admiration and rivalry and two self-esteem-related metrics at the group level. The thresholds are a “proof of concept” and intended to demonstrate that the underlying traits between groups in simulated and EMA data are similarly distributed. The metrics of median and variance of self-esteem were calculated for each participant or agent. While we depict the values of each individual for the EMA data, we report only the group mean for the simulated data, as we can exclude noise due to the strict model specifications.

## Results

### Model of self-esteem regulation and learning environment

The core of our self-esteem regulation (SER) model is the interaction of self-esteem and inter-individual self-esteem regulation in narcissism. It features two levels: agent-internal model of self-regulation and external model of agent interactions. A comprehensive description of these model components and associated mechanisms is provided in Fig. 1.

For the internal model (Fig. 1, blue area), self-esteem and need for admiration are the fundamental components and their interaction drives self-esteem regulation. When an agent’s self-esteem deviates significantly from the need for admiration, this triggers one of three types of behaviors for self-esteem regulation. The first of these behaviors is the intra-individual regulation of self-esteem. The other two behaviors use inter-individual interactions, namely admiration and rivalry. An agent chooses between these behaviors based on thresholds which reflect its predisposition towards these behaviors. This predisposition varies between agents, leading to differing probabilities of exhibiting any of these behaviors. The internal self-regulation behavior provides the agent with a deterministic but relatively low amount of self-esteem boost. In contrast, the inter-individual behaviors, admiration and rivalry, depend on interactions with agents in the environment, which is provided by the external model.

The external model (Fig. 1, purple area) consists of agents that interact with each other and may respond differently to the self-esteem regulation attempts by each other agent. Positive responses elevate the self-esteem of the other agent, while negative responses lower the self-esteem of the other agent. Whether an agent chooses to respond positively or negatively depends on two influences: first, on the type of inter-individual self-regulation behavior shown by the other agent, with admiration having a higher chance of positive responses compared to rivalry; second, on the relative self-esteem of both agents.

Additionally, to the direct effects of self-esteem regulation, the model incorporates two mechanisms of adaptation. First, the need for admiration habituates to the agent’s level of self-esteem, leading to an ever increasing or decreasing need for admiration reflecting a tolerance development akin to the tolerance development in substance addiction^30^.

The second adaptation mechanism is a reinforcement learning component (Fig. 1, gray area), which changes an agent’s predisposition towards the inter-individual self-regulation behaviors. Depending on receiving positive or negative responses, an agent reinforces its predisposition positively or negatively. This learning component links the contingencies of the external model and the predispositions in the agent-internal model.

This learning component may be active or not, providing two distinct modes. First, a basic SER model in which a preconfigured agent acts based on its unchanging predispositions. This enables the study of an “adult”, narcissistic agent acting on once acquired predispositions in a stable everyday environment. Second, the learning SER (LSER) model in which a learning agent acts based on its adapting predispositions. This enables the study of “growing up”, naive agents, acquiring predispositions in different environments which could for example reflect different parenting styles.

### Study 1: inter-individual self-esteem regulation and self-esteem

**Fig. 2:**
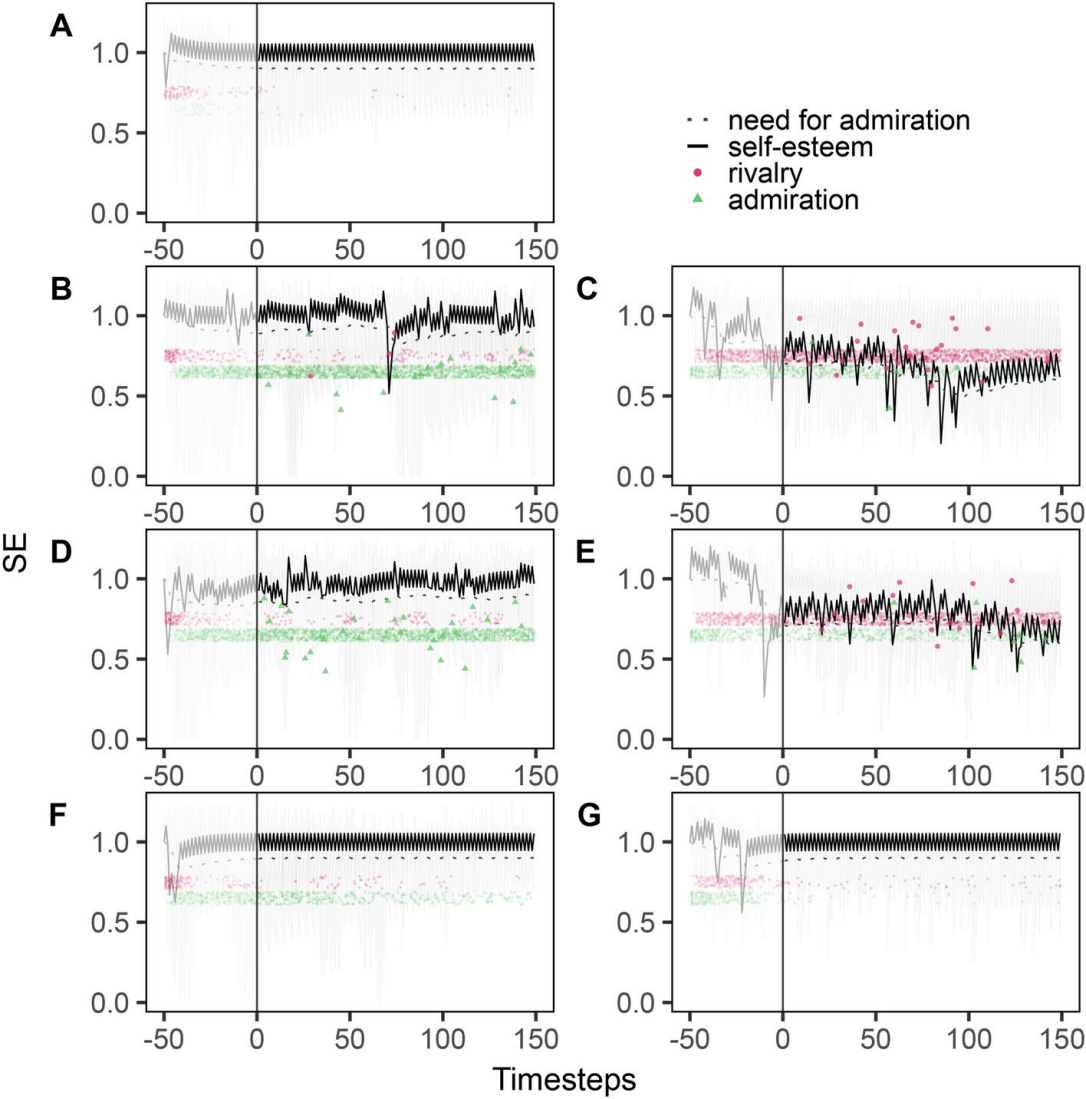
Simulation of Self-esteem (SE), need for admiration (NfA), and self-esteem regulation behavior (admiration and rivalry) for different thresholds to initialize admiration (TS_admi_) or rivalry (TS_riva_). (**A**) Threshold default values with TS_admi_ = 0.08, TS_riva_ = 0.09. (**B**, **D**, **F**) Variation of admiration threshold (TS_admi_) while keeping the rivalry threshold (TS_riva_) at its default value, with TS_admi_ = 0.0055 (**B**), TS_admi_ = 0.025 (**D**), and TS_admi_ = 0.05 (**F**). (**C**, **E**, **G**) Variation of rivalry threshold (TS_riva_) while keeping the admiration threshold (TS_admi_) at its default value, with TS_riva_ = 0.0055 (**C**), TS_riva_ = 0.025 (**E**), and TS_riva_ = 0.05 (**G**). (**A**–**G**) The transparent lines and points depict all agents’ self-esteem (solid line) and regulation behavior (admiration: green triangle, rivalry: red point); the non-transparent elements depict a prototypical agent’s self-esteem (solid line), need for admiration (dotted line), and regulation behavior (admiration: green triangle, rivalry: red point). The simulation was run with a burn-in period of *Δt* = 50 and with n = 50 agents each.

With our first simulation study, we aimed at gaining a deeper understanding of the association between regulation behavior and self-esteem oscillations. Following the narcissism admiration and rivalry (NARC) theory^28,29^, narcissism is characterized by self-idealizing (admiration) and other-devaluing (rivalry) behavior which both require inter-individual interactions. Hence, we first focused on inter-individual self-esteem regulation and tested varying thresholds to activate the self-esteem regulation behaviors admiration and rivalry (i.e., values of 0.0055 [low; Fig. 2b,c], 0.025 [medium; Fig. 2d,e], and 0.05 [high; Fig. 2f,g], where lower values represent lower thresholds).

Our results show that for both thresholds, admiration and rivalry, lower values are associated with stronger oscillations in self-esteem and more frequent self-esteem regulation with the respective behavior, i.e., more admiration with a lower admiration threshold and more rivalry with a lower rivalry threshold. Occasionally, agents also use the behavior with default threshold (e.g., Fig. 2b. which shows a sporadic display of rivalry even though only the admiration threshold was lowered).

The two distinct thresholds (admiration and rivalry) also show differential patterns. Lower values of the admiration threshold lead to higher levels of self-esteem and more upward spikes while oscillations are less pronounced (e.g., self-esteem spike at *t* = 125 timesteps in Fig. 2b). In comparison, lower values of the rivalry threshold lead to lower self-esteem levels and more drops, while oscillations are stronger (e.g., a massive drop in self-esteem at *t* = 75 timesteps in Fig. 2c).

Overall, more escalating behavioral patterns are observed as the threshold values for activating behavior decrease. At the same time, diverging patterns emerge for low admiration thresholds (oscillations at a higher level with occasional spikes and drops) and low rivalry thresholds (strong oscillations at a lower level with repeated drops). Hence, diverging narcissistic behaviors are observable depending on an agent’s predisposition.

### Study 2: simulation experiments in the learning environment

Study 1 showed that an agent’s predisposition has distinct influences on typical behavioral patterns of narcissistic self-esteem regulation. With our second simulation study, we aimed at investigating how the learning environment shapes this predisposition. Therefore, we ran the LSER model (with activated learning component) in three different environments (i.e., overvaluing and praising, devaluing and indifferent, control environment) and observed the thresholds to activate self-esteem regulation behavior as well as the self-esteem course over time.

**Fig. 3:**
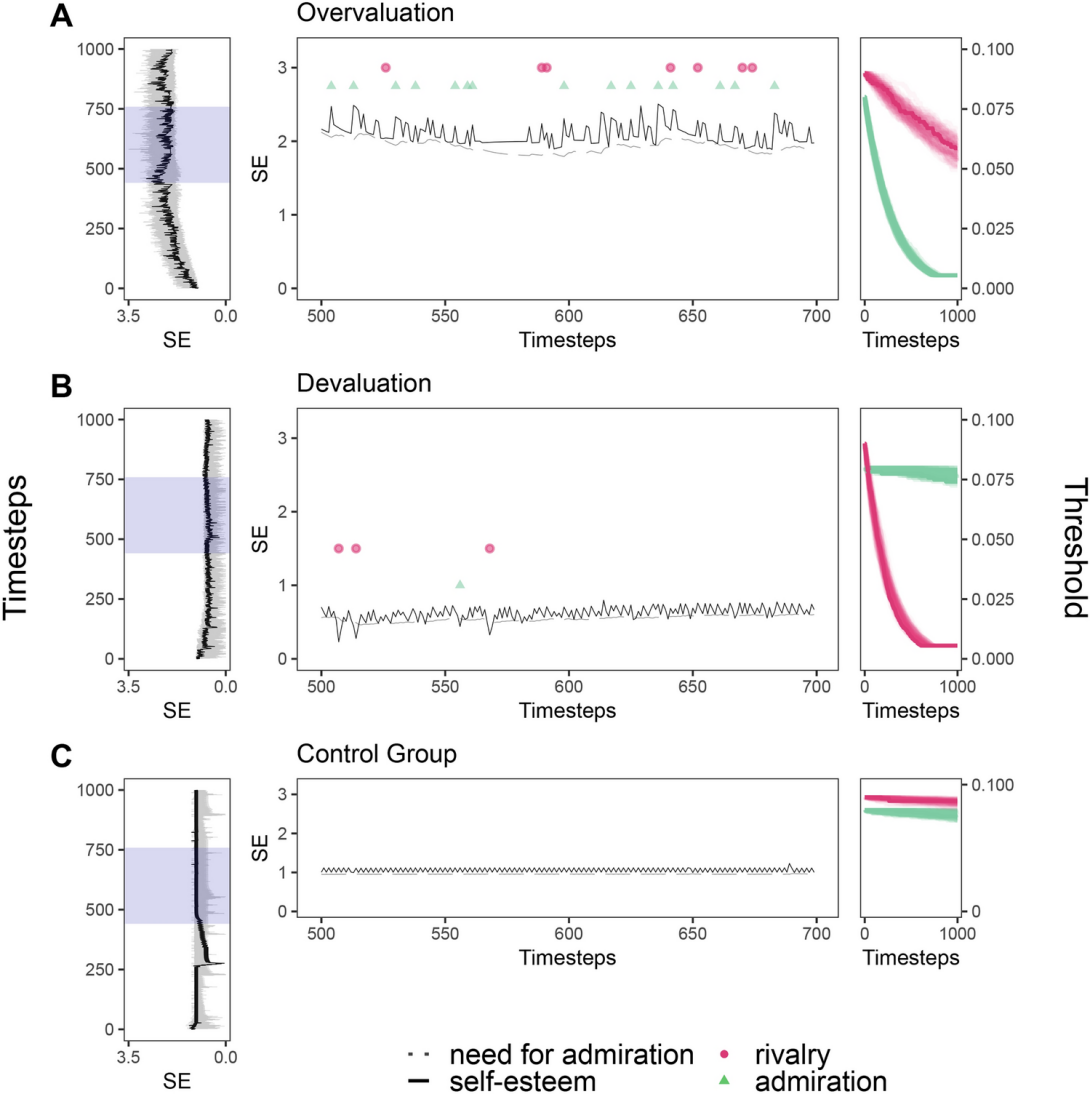
Simulation of self-esteem (SE), need for admiration, self-esteem regulation behavior (admiration and rivalry), and thresholds to initialize admiration (TS_admi_) and rivalry (TS_riva_) for agents who learn self-esteem regulation behavior in three different environments. (**A**) Overvaluing and praising environment, characterized by highly positive contingent and non-contingent feedback to an agent. (**B**) Devaluing and indifferent environments with indifferent contingent and negative non-contingent feedback to an agent. (**C**) Control environment with behavior-oriented contingent and almost none non-contingent feedback. (**A**–**C**) The self-esteem course (left) and the learning process of threshold values (right) for rivalry (red) and admiration (green) are depicted for n = 50 agents over 1000 timesteps. One prototypical agent is highlighted (black solid line in the left-side plots) and depicted in more detail for the timestep interval t[500; 700] marked by the blue shaded area on the left-side plots. The central plots depict one prototypical agent’s self-esteem course (solid line), need for admiration (dotted line), and self-esteem regulation behavior in the form of admiration (green) and rivalry (red).

Our results show that in the overvaluing and praising environment the threshold to activate admiration decreased substantially while the threshold for rivalry decreased only slightly (see Fig. 3a). Self-esteem oscillates marginally at a very high level and is recurrently regulated by admiration and rivalry. Since the values of the need for admiration develop relative to absolute self-esteem values, we repeatedly observe relatively small, but absolutely large differences between self-esteem and need for admiration which exceed the rivalry threshold and therefore cause the frequent rivalrous self-esteem regulation.

In the devaluing and indifferent environment, the threshold to activate rivalry decreased to values close to the predefined lower boundary, while the admiration threshold changed only slightly (see Fig. 3b). The self-esteem remains mostly stable at a low level while agents regulate self-esteem rarely with rivalry on an inter-individual level. In the control environment, both thresholds showed only minor variations and self-esteem varies barely while almost no inter-individual self-esteem regulation is shown (see Fig. 3c).

In summary, in the LSER model, non-contingent feedback has a strong influence on the direction and extent of learning, while contingent feedback shows more subtle influences. Contingent feedback both affects the temporal dynamics of learning as well as the variation of the learned thresholds across agents. Hence, a non-contingent, overvaluing environment shapes a predisposition that results in behavioral patterns of narcissistic self-esteem regulation that is associated with grandiose narcissism (i.e., more frequent admiration regulation and higher self-esteem levels) while a non-contingent, devaluing environment shapes a predisposition that results in a behavioral pattern that is associated with vulnerable narcissism (i.e., more frequent rivalry regulation and lower self-esteem levels). Regarding the self-esteem courses over time, we see regular oscillations in all three environments without significant spikes or drops. Although narcissistic behavior in the form of inter-individual self-esteem regulation is learned, our findings do not indicate the clear association between these behaviors and irregular self-esteem oscillations with recurrent spikes and drops as demonstrated in Study 1 as long as the environmental conditions remain constant.

### Study 3: simulation experiments with learned agents

Study 2 showed that the predispositions to activate inter-individual regulation behavior are strongly affected by non-contingent environmental feedback. With our third simulation study, we aimed at understanding how these self-esteem dynamics evolve when an agent with a learned predisposition from overvaluing and praising environments or devaluing and indifferent environments is exposed to regular environments (i.e., with behavior-related and only contingent feedback). Therefore, we ran the SER model with the average learned thresholds from Study 2, exposed all agents to the control environment, and we observed individual variations in the self-esteem as well as regulation behavior frequencies.

**Fig. 4:**
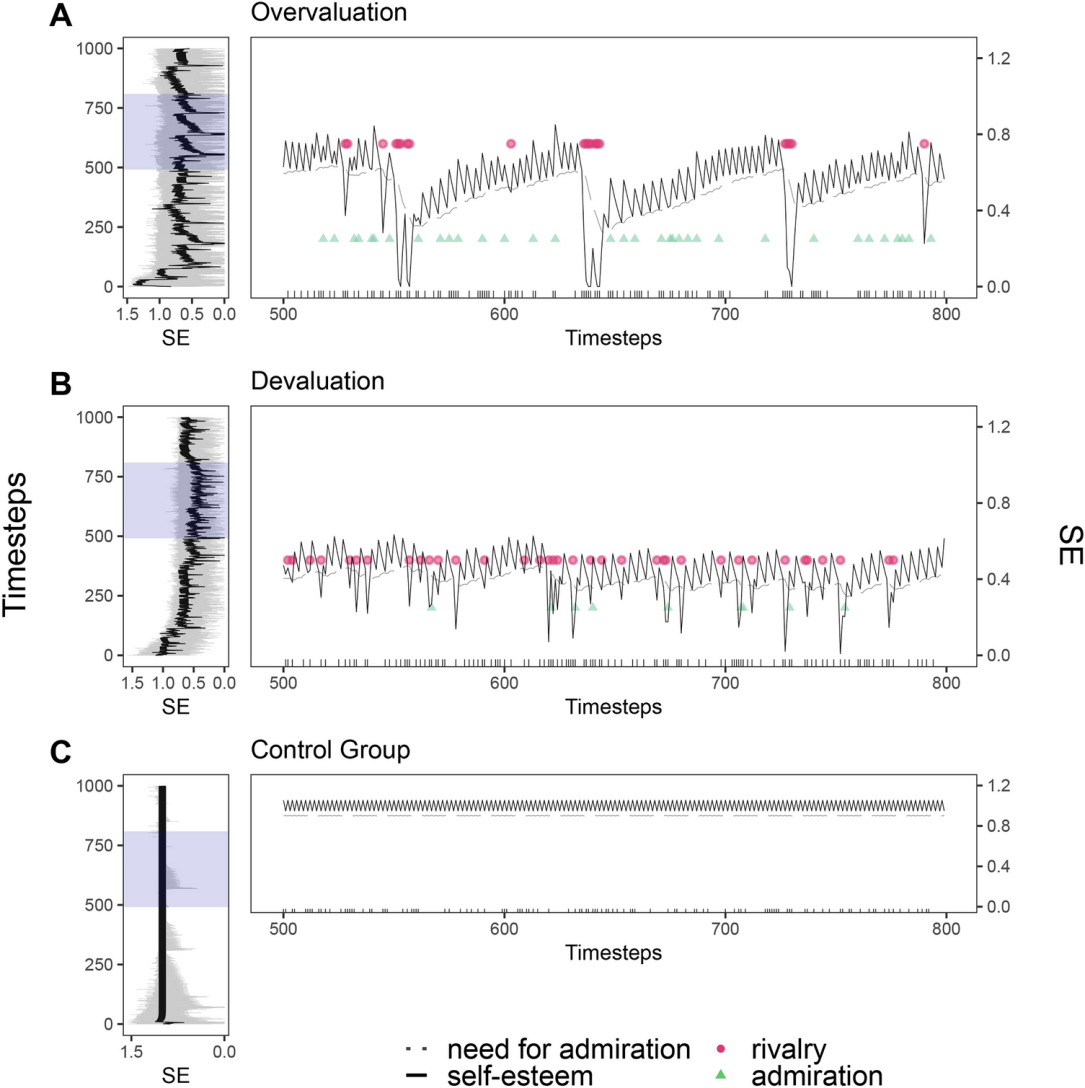
Simulation of self-esteem (SE), need for admiration, self-esteem regulation behavior (admiration and rivalry), and whether an agent is alone or together with other agents for agents who have learned their regulation predisposition (i.e. thresholds for initializing admiration or rivalry) in three different environments. (**A**) Agents learned in an overvaluing, praising environment. Initial model values for admiration and rivalry thresholds: TS_admi_ ~ N(0.0055, 0.0); TS_riva_ ~ N(0.0576, 0.0037). (**B**) Agents learned in a devaluing, indifferent environment. Initial model values for admiration and rivalry thresholds: TS_admi_ ~ N(0.0833, 0.0029); TS_riva_ ~ N(0.0072, 0.0007). (**C**) Agents learned in a control environment. Initial model values for admiration and rivalry thresholds: TS_admi_ ~ N(0.0562, 0.0037); TS_riva_ ~ N(0.1125, 0.0074). (**A**–**C**) The self-esteem course on the left side is depicted for n = 50 agents over 1000 timesteps. One prototypical agent is highlighted (black solid line in the left-side plots) and depicted in more detail for the timestep interval t[500; 800] marked by the blue shaded area on the left-side plots. The central plots depict one prototypical agent’s self-esteem course (solid line), need for admiration (dotted line), self-esteem regulation behavior in the form of admiration (green) and rivalry (red), and whether an agent is alone or together with other agents. A black vertical line on the x-axis in the central plots indicates that other agents (i.e., potential interaction partners, are present).

Our results show distinct individual variations in the self-esteem for agents from the overvaluing and praising environment, agents from the devaluing and indifferent environment, and agents from the control environment (see Fig. 4). Agents that learned their predisposition in an overvaluing and praising environment show a generally high level of strongly oscillating self-esteem. They mostly use admiration and regulate their self-esteem just occasionally with rivalry, which often fails and leads to self-esteem drops (see *t* = 550 timesteps; see Fig. 4a). Strong self-esteem spikes are typically followed by equally strong drops. Agents reveal a pattern of self-esteem regulation that can be described as tolerance development, that is, there is a gradual adjustment of the need for admiration over time. The regularly occurring regulation via admiration causes an increased need for admiration (see *t* = 770 timesteps), which mimics a need for recurring self-enhancement^30^. This increases the risk that the environmental feedback does not meet the entitled expectation, leading to self-esteem drops (see *t* = 780 timesteps). Agents that learned their predisposition in a devaluing and indifferent environment show a generally low level of oscillating self-esteem. They mostly use rivalry as regulation behavior which often leads to self-esteem drops (see Fig. 4b). This pattern is only interrupted when an agent no longer finds an interaction partner (see* t* = 780 timesteps). Agents reveal a pattern of self-esteem regulation which can be described as a vicious cycle of inter-individual behavior. Their frequent use of rivalry goes along with a high probability of negative responses from the environment and hence a failed regulation of self-esteem which, in turn, leads to even more need for self-esteem regulation and hence new attempts of rivalry. This vicious cycle mimics the ongoing decline in self-esteem when agents persist in dysfunctional behavior^26,38^. This pattern can also be found in overvalued agents, e.g., at *t* = 550 timesteps. Agents that learned their predisposition in the control environment exhibit minor oscillations and a consistently high level of self-esteem (see Fig. 4c), similar to the baseline condition in Study 1 (Fig. 2a).

In summary, agents that did not learn in the control environment show strong self-esteem oscillations and frequent use of inter-individual regulation behavior. Agents from an overvaluing and praising environment exhibit strong self-esteem oscillations on a high level while regulating with admiration. Repeated use of rivalry leads to recurrent drops in self-esteem. Agents from a devaluing and indifferent environment exhibit self-esteem oscillations on a low level, regulating mostly with rivalry. Two patterns of self-esteem regulation emerge: tolerance development (i.e., adapting to positive environmental responses resulting in an increased need for admiration and therefore facing the risk of not being satisfied) and a vicious cycle of inter-individual behavior (i.e., frequent use of rivalry). Both patterns are associated with strong oscillating self-esteem and frequent inter-individual regulation, hence potentially escalating behavioral patterns. This demonstrates that a change in the environment can lead to a mismatch between learned narcissistic behavior and the current requirements of the environment.

### Study 4: replication of real world data with simulated data

Study 4 aimed to probe the external validity of the model. (e.g., oscillating self-esteem) from real world data in simulated data. To this end, we synchronized simulated data with real world data from an ecological momentary assessment study (EMA^4^), where participants were categorized into four groups based on their scores in vulnerable and grandiose trait narcissism: (1) low grandiose, low vulnerable (LG,LV), (2) high grandiose, low vulnerable (HG,LV), (3) low grandiose, high vulnerable (LG,HV), (4) high grandiose, high vulnerable (HG,HV) narcissism.

**Fig. 5:**
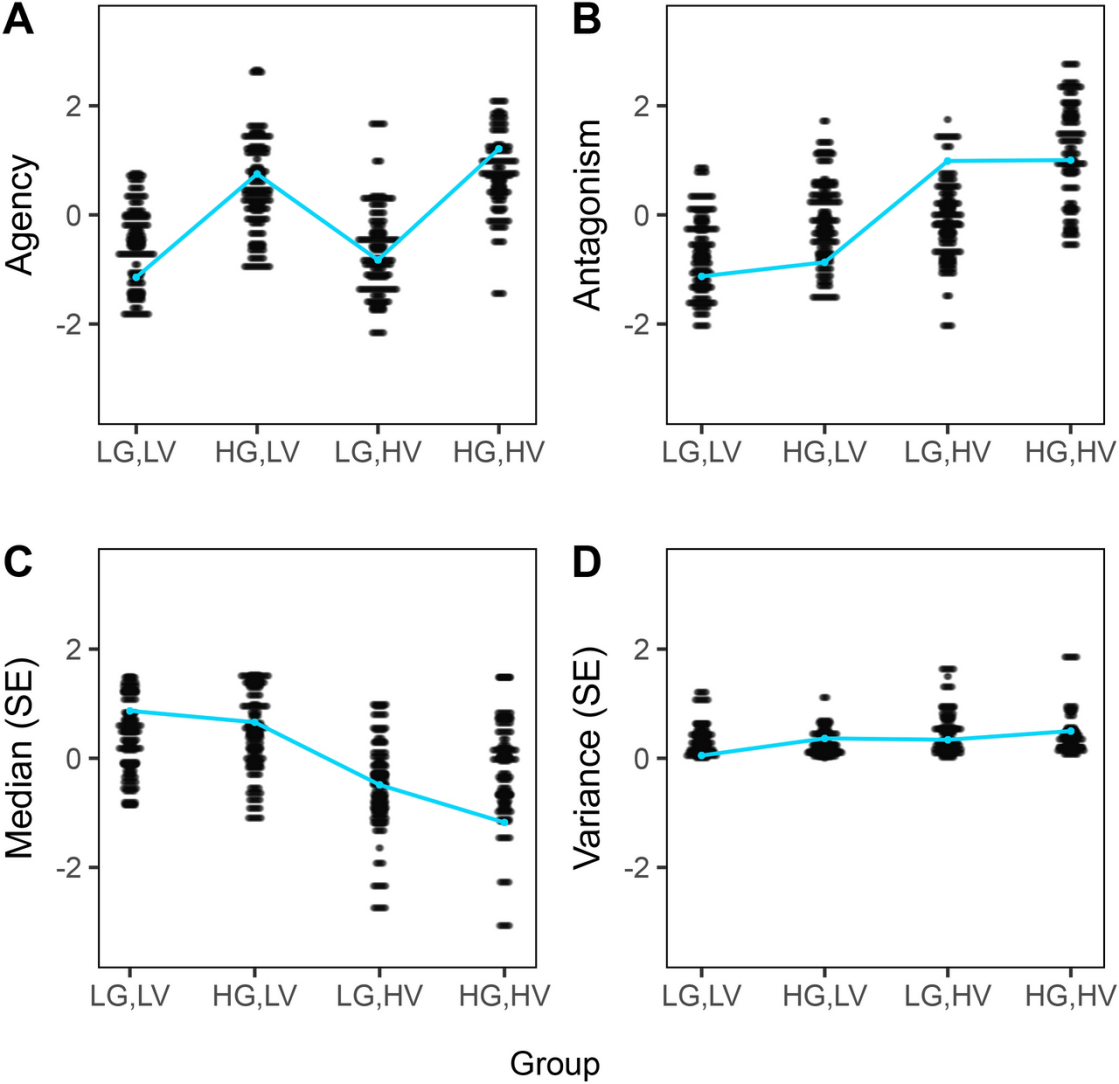
Group-level self-esteem regulation behavior (agency/admiration and antagonism/rivalry) and self-esteem (SE) related values (z-standardized) for EMA (one data point per subject; N = 167) and simulated data (group level means, N = 200). (**A**) Values of FFNI agency (EMA) and inverted (value * − 1) mean threshold value of all groups to initialize admiration (simulated data) data, z-standardized. (**B**) Values of FFNI antagonism (EMA) and inverted (value * − 1) mean threshold value of all groups to initialize rivalry (simulated data), z-standardized. (**C**) Median of single self-esteem courses over time, z-standardized. (**D**) Variance of single self-esteem courses over time, z-standardized. (**A**–**D**) EMA data was categorized into four groups based on NPI and HSNS measured traits, model data was simulated according to this categorization. This resulted in the four depicted groups, namely (LG,LV) low grandiose and low vulnerable (n_ema_ = 42, n_sim_ = 50), (HG,LV) high grandiose, low vulnerable (n_ema_ = 46, n_sim_ = 50), (LG,HV) low grandiose, high vulnerable (n_ema_ = 42, n_sim_ = 50), (HG,HV) high grandiose, high vulnerable narcissism (n_ema_ = 37, n_sim_ = 50). The FFNI values are traits (i.e., the higher the value, the higher the trait expression) which are conceptualized in the model as thresholds (i.e., the lower the values, the higher the trait expression). For this reason, the mean values of the FFNI scores from the EMA data were inverted and adjusted to match the possible value range of the simulated data (i.e., the highest value in the FFNI data equals the lowest threshold value in the simulated data).

Our replication efforts focused on four variables as depicted in Fig. 5: *agency*/*admiration* which represents grandiosity and self-presentation, *antagonism/rivalry* which represents entitlement and aggressive and derogating behavior towards others, the *self-esteem median* which represents the median of the self-esteem course over time of each individual and the *self-esteem variance* which represents the variance of each individual self-esteem course over time of each individual.

For agency, both simulated and EMA data show comparatively low values in Group *LG,LV* and Group *LG,HV*, compared to higher values in Group *HG,LV* and Group *HG,HV* (Fig. 5a). For antagonism, the data show a similar rising trend in both data sets with the difference that the EMA data show a gradual increase across all groups, while the simulated data show a more distinct jump between Groups *HG,LV* and *LG,HV* (Fig. 5b). For self-esteem median, both data sets show similar patterns (Fig. 5c): While the values are high in Group *LG,LV* and Group *HG,LV*, they decrease in Groups *LG,HV* and Group *HG,HV*. For self-esteem variance, the data show an almost linear increase with Group (Fig. 5d).

## Discussion

Conceptualizing narcissism is complex: Diverging etiological theories intersect with various descriptions of phenotypical behavior. As an approach to identify hidden causal mechanisms behind patterns of narcissistic behavior observed in the real world, we used agent-based modeling and investigated assumptions on the genesis of narcissistic self-esteem regulation. We designed a theory-driven, computational model for narcissistic self-esteem regulation behavior and their associated learning processes. Across three simulation studies, we showed that inter-individual self-regulation behavior such as admiration and rivalry are linked to self-esteem oscillations and are primarily adapted through a non-contingent over- or devaluation in the environment, which leads to four central insights.

First, the thresholds for activating inter-individual regulatory behaviors, admiration and rivalry, cause narcissism-typical strong oscillations in self-esteem. When the threshold for admiration is lower and admiration is hence more frequently employed as self-esteem regulation behavior, then the model shows reliable self-esteem oscillations at a high level. Conversely, when the threshold for rivalry is lower and rivalry is hence primarily used as a regulation strategy, then the model shows strong self-esteem oscillations at a lower level. Both forms of inter-individual self-regulation behavior are empirically and conceptually linked to distinct manifestations of narcissism. While admiration (i.e., confident entitlement and self-presentation) is associated with agentic strategies^55^, rivalry (i.e., hostile devaluation of others) is tied to antagonistic strategies in narcissism^55^. Agentic narcissism is characterized by moderate self-esteem oscillations on a high level^26^. In contrast, antagonistic narcissism is associated with strong self-esteem oscillations on a low level^26^. Corresponding patterns of self-esteem emerge in our mode, although our model was not built with a specific representation of self-esteem oscillations and associated regulation strategies. Even if just single threshold values change (i.e. either admiration or rivalry threshold), both regulation behaviors are shown more frequently, especially for the lowest threshold values.

Second, the two inter-individual self-regulation behaviors show distinct patterns of tolerance development for admiration and a vicious cycle for rivalry. These result in escalating behavioral patterns, characterized by more frequent displays of inter-individual self-esteem regulation followed by adverse social consequences. Admiration is mostly followed by a substantial rise in self-esteem. This leads to tolerance development in self-esteem where agents need more and more appreciation which manifests in an increase of the expectancy value of external feedback. The environment cannot meet this increased expectation of recurrent self-enhancement (cf. “addiction to esteem”^30^). Hence, the rise in self-esteem following admiration triggers a more frequent occurrence of inter-individual self-regulation behaviors: more admiration, and sometimes even rivalry. In contrast to this, rivalry is mostly followed by drops in self-esteem. Periods of consecutive rivalry regulation hence recurrently coincide with a decline in self-esteem toward a lack of self-esteem which only may recover in the absence of interaction partners. This vicious cycle of rival self-esteem regulation with constantly decreasing self-esteem, where time alone seems helpful to recover self-esteem, is in line with the evidence for highly contingent self-esteem in terms of interpersonal experiences (cf.,^26,55–57^). Without social interaction, self-esteem is less dependent on social feedback and can be counter-regulated intra-individually.

Third, overvaluing and praising environments cause high-level oscillating self-esteem patterns with repeated self-esteem drops. While self-esteem is mostly regulated with admiration, agents periodically regulate with rivalry (see Fig. 4a, t = 750 timesteps), coinciding with a significant and abrupt decrease in self-esteem. In contrast, devaluing and indifferent environments cause strong low-level self-esteem oscillations and the frequent use of rivalry as a regulation strategy (cf.,^26,58,59^). The self-esteem course of the overvalued agent represents a combination of grandiose and vulnerable dynamics (e.g.,^60^). Considering findings on the non-linear concatenation of grandiose and vulnerable states, this can be classified as pathological narcissism^6^. Accordingly, in highly grandiose expressions, stronger vulnerable components are found, which Morf and Rhodewalt^24^ view as a logical consequence of grandiosity: If one’s self-view is that “puffed up” and one’s sense of entitlement is that high^26^, there are many possibilities to attack and frustrate these perceptions. This dynamic of recurrent strong collapses of self-esteem inherently embodies narcissistic processes such as striving for self-enhancement and entitlement rage^61^. On the contrary, the self-esteem pattern of the devalued agent mirrors a general rather than specific narcissistic pathology^62^. Based on findings of increased impulsivity, emotional dysregulation, and rejection sensitivity, analogies between vulnerable narcissism and borderline personality are discussed^63–65^. Growing up in a devaluing environment can be seen as a general risk factor for the deprivation of certain basic needs such as attachment and appreciation, thus it can be seen as pathology-promoting^66^.

Fourth, high grandiose and high vulnerable narcissism are associated with dynamic shifts of grandiose and vulnerable self-esteem patterns in simulated and empirical data. Periods of high-level self-esteem with mostly admiration regulation alternate with self-esteem drops and frequent rivalry regulation, reflected in high variance of single self-esteem courses (cf. Fig. 5d). Hence, these shifts between grandiose and vulnerable states reflect a potentially dysfunctional pattern of narcissistic self-esteem regulation marked by self-esteem instability and rigid self-esteem regulation patterns^49,67^. Rigid behavior may not be adaptable to changing situations, resulting in a mismatch of interaction behavior and contextual requirements. The interplay of the model with different contextual requirements (e.g., a “childhood” and an “adult” environment) indicates that the adaptivity of behavior depends on both environmental (i.e., positive vs. negative feedback) and interactional characteristics (i.e., regulating self-esteem with admiration vs. rivalry). Hence, the stability of self-esteem hinges on the alignment between contextual and individual characteristics.

With these results, we present an approach to understand self-esteem regulation in narcissism as a complex system. This allows us to depict contrary processes at different time scales, such as the long-term acquisition of narcissism-typical traits (etiological genesis of narcissism) and the short-term adaptation of self-esteem through inter-individual behavior to reduce the aversive need for regulation while potentially causing adverse social effects in the longer run in narcissism^68^. These linkages cannot be assessed with static methods. Particularly with regard to the development of prevention and intervention methods of dysfunctional behavior, we should understand human behavior as a complex system that needs to be addressed with appropriate methods^69,70^. To our knowledge, there is no dynamic process model that covers and links all of these components in narcissism. Therefore, our model could serve as a groundwork for theory building and identification of causal processes in the formation of narcissistic behavior. As an example, our model already provides initial insights into potential interventions in cases of highly frequent self-esteem regulation with rivalry which is often associated with significant drops in self-esteem (see Fig. 4a, t = 550 timesteps). There are two possible ways out: accept the low self-esteem level or social isolation. Agents could get used to the low self-esteem level and thus have no longer the need for regulation^30^. Social isolation, as depicted at *t* = 780 timesteps, means that agents are at distance from potential interaction partners, allowing agents to change back to intra-individual regulation which increases self-esteem (cf.,^27,56^).

The resulting self-esteem-related metrics display similar patterns at the group level in both simulated and real world data. This points out that the model might depict (and potentially explain) real world patterns, even though we cannot estimate the temporal resolution of the model. At the same time, the values also diverge strikingly at some points (e.g., TS_riva_ in Fig. 5b). On the one hand, this might be due to the ideal–typical, theory-driven focus of the model. For instance, the simulated values of the control group (low vulnerable, low grandiose) predominantly exhibit regular, very small oscillations, with entropy values close to 0. In contrast, the EMA data is far from ideal–typical regarding theoretical assumptions. We see more noise and the variance within single groups is occasionally vast. Upon closer examination of individual self-esteem courses in the EMA data (see Supplementary Figs. S1–S4), it becomes evident that both vulnerable states (i.e., low-level self-esteem oscillations) and grandiose states (i.e., higher level self-esteem oscillations) are present across all subgroups. This aligns with the understanding of narcissism as a spectrum. Vulnerable and grandiose states can emerge in all trait groups, with the likelihood varying depending on the specific characteristics on a trait level (i.e., in individuals with a trait-level narcissistic vulnerability, there is a higher probability of experiencing vulnerable states). The high variance in EMA data can therefore also be interpreted as a call to deviate from rigid categorization and to focus on narcissism as a dynamic process. It remains unclear when grandiose or vulnerable states manifest and what factors influence this occurrence. What triggers a transition between these states? The model falls short in capturing reality in these aspects due to its lack of complexity in encompassing potential influencing factors. Similarly, the rigid, trait-based categorization in the EMA data does not adequately address the dynamic processes at the state level.

Despite the promising results, the presented results are only one modeling approach, which might be “wrong but useful”. Several open questions remain, of which the following four seem particularly relevant to us. First, the conceptualization and operationalization of intra-individual regulation should be explored in more detail to provide a deeper understanding of the meaning of this strategy^71^. Second, the model primarily focuses on the intrapsychic consequences of inter-individual behavior, specifically how it influences the self-esteem of agents. Therefore, the model does not adequately account for the temporal evolution of relationships between individual agents, nor does it adequately describe the long-term social consequences of problematic inter-individual dynamics. Implementing social relations within the model would represent a promising avenue for future research. Third, it is important to systematically test the implemented threshold assumptions. While the NARC posits a hierarchical structure of admiration and rivalry (first regulation with admiration, if this does not work rivalry), we decided to design our model to be more flexible and enable lower admiration than rivalry thresholds. The effects of the different threshold values and the sequential order should be tested systematically, such as, how the model behavior changes depending on fixed lower admiration, fixed lower rivalry or flexible thresholds. In order to build a relatively simple first model, we decided to implement fixed thresholds to the SER model. It should be tested how the model behavior changes with flexible thresholds, so agents can continuously learn to a certain extent. Fourth, conducting a systematic simulation experiment to examine the impact of environmental factors on the transition between admiration and rivalry could be an intriguing avenue for future research. Using the SER model, we could analyze in-depth the relationship between environmental responses to the displayed regulation behavior and the associated mechanisms behind changing self-esteem regulation strategies. Our models may provide the framework for identifying further critical contextual parameters and regulation mechanisms and deepening our understanding of the included dynamics to ease the process of operationalizing and testing assumptions. Such critical tests could, for example, target the two postulated main mechanisms, tolerance development and the vicious cycle of rivalry, which we expect to be interruptible by phases of social isolation.

Based on these two mechanisms, our findings provide suggestions for preventive and therapeutic interventions to enhance overall personality functions and targeting extreme patterns of self-esteem regulation. Therapeutic interventions should focus on two main goals: strengthening intra-individual regulation competencies and mitigating the negative social consequences of hostile inter-individual self-esteem regulation. Our results can be interpreted as an indication that, inter alia, both cognitive-behavioral (e.g., structured training to internalize self-affirming beliefs) and psychodynamic therapeutic approaches (e.g., fostering new interactional experiences with the therapist and transfer these into daily life) may be effective in strengthening intra-individual self-regulation skills^72,73^. For mitigating negative social consequences, the mechanisms of tolerance development and the vicious cycle of rivalry provide valuable insights for therapeutic interventions. Understanding the dynamics of tolerance development enables early intervention before a self-esteem crash occurs. Therapeutic efforts could focus on fostering a realistic perspective on feedback from the environment, reducing the risk of a detrimental mismatch between inflated expectations of external validation and the actual feedback received from others (e.g., interventions to enhance social cognition might be helpful; cf.^4^). Additionally, the model shows that phases of repeated rivalrous, hostile behavior can be buffered through selective periods of solitude. Therapeutically, it could be valuable to help individuals identify situations where withdrawing into intra-individual self-regulation is beneficial, instead of resorting to demeaning, hostile, or hurtful behavior, which often leads to negative long-term consequences such as damaged relationships, friendships, or professional ties^74^.

The results of the learning model suggest a preventive approach, emphasizing that young individuals should learn to develop resilient self-esteem regulation mechanisms. This includes fostering a realistic evaluation of their own behavior, internalizing positive parental feedback, and ultimately providing the fundament for intra-individual self-regulation. The modeling results imply that this can best be achieved through a realistic link between actions and consequences—via contingent feedback—ensuring that children do not become dependent on non-contingent external feedback. However, the model only offers preliminary insights, and more simulation studies and real-world research should test specific prevention and intervention strategies.

In summary, our findings indicate that grandiose narcissistic self-esteem regulation, characterized by the frequent use of admiration and rivalry as inter-individual self-esteem regulation strategies, predominantly develops in overvaluing and praising environments. When these learned self-regulation strategies are subjected to a change in environment, two typical self-esteem patterns as maintaining mechanisms emerge: tolerance development which reflects assumptions of narcissism as “addiction to esteem”^30^ and a vicious cycle of rivalry which reflects the broad evidence on interactional difficulties in narcissism^8,55^. Shifting from static to dynamic analysis through agent-based modeling, our model provides critical insights for the early detection of escalating self-esteem patterns, suggesting possible interventions such as social time-outs or the intentional withdrawal” of social admiration. Our work offers an experimental space for generating and testing causal hypotheses on the etiology of narcissistic self-esteem regulation. As a formalized proof of concept for established etiological theories, our model shows that parenting styles indeed provide a learning environment in which narcissistic behavior can emerge. With this information on the “why” of behavior, we may initiate a transfer to a more precise understanding of underlying mechanisms, i.e., the “how” of behavior, and start to test candidates for successful intervention. These steps become even more relevant given the debates of increasing narcissistic behaviors through socio-cultural influences (cf.,^75^) and the association of stable self-esteem with fundamental healthy psychological functions^76–79^.

## Supplementary Information

Supplementary Information.

## Data Availability

The model and analysis code as well as data of the simulation studies is available at https://osf.io/ca5dn/. Data from the ecological momentary assessment study is available at https://osf.io/jdwav/.
