# Supplementary material for: A computational framework to study the etiology of grandiose narcissism
Source: Sci Rep. 2025 Feb 18;15:5897. doi: 10.1038/s41598-025-90109-w (PMC11836455; doi:10.1038/s41598-025-90109-w)
Supplement: Supplementary file 1 — Supplementary Information. [file 41598_2025_90109_MOESM1_ESM.pdf]

Supplementary Information (SI)

A computational framework to study the etiology of grandiose narcissism

Deborah M. Löschner[1], Martin Schoemann[2], Emanuel Jauk[3], Lena Herchenhahn[4], Sarah Schwöbel[5], Philipp Kanske[5], Stefan Scherbaum[2]

[1] Institute of Work, Organisational and Social Psychology, Technische Universität Dresden, 01069 Dresden, Germany
[2] Institute of General Psychology, Biopsychology and Methods of Psychology, Technische Universität Dresden, 01069 Dresden, Germany
[3] Department of Medical Psychology, Psychosomatics, and Psychotherapy, Medical University of Graz, 8036 Graz, Austria
[4] Institute of Psychology, Christian-Albrechts-Universität zu Kiel, 24118 Kiel, Germany
[5] Institute of Clinical Psychology and Psychotherapy, Technische Universität Dresden, 01187 Dresden, Germany

***Correspondence:**
Deborah Löschner
deborah_maria.loeschner@tu-dresden.de

Keywords: etiology, narcissism, agent-based modeling, computational psychology, personality

This supplementary information file contains:

- SI A: Supporting Figures
- SI B: Supporting Tables
- SI C: Supporting Text

SI A: Supporting Figures

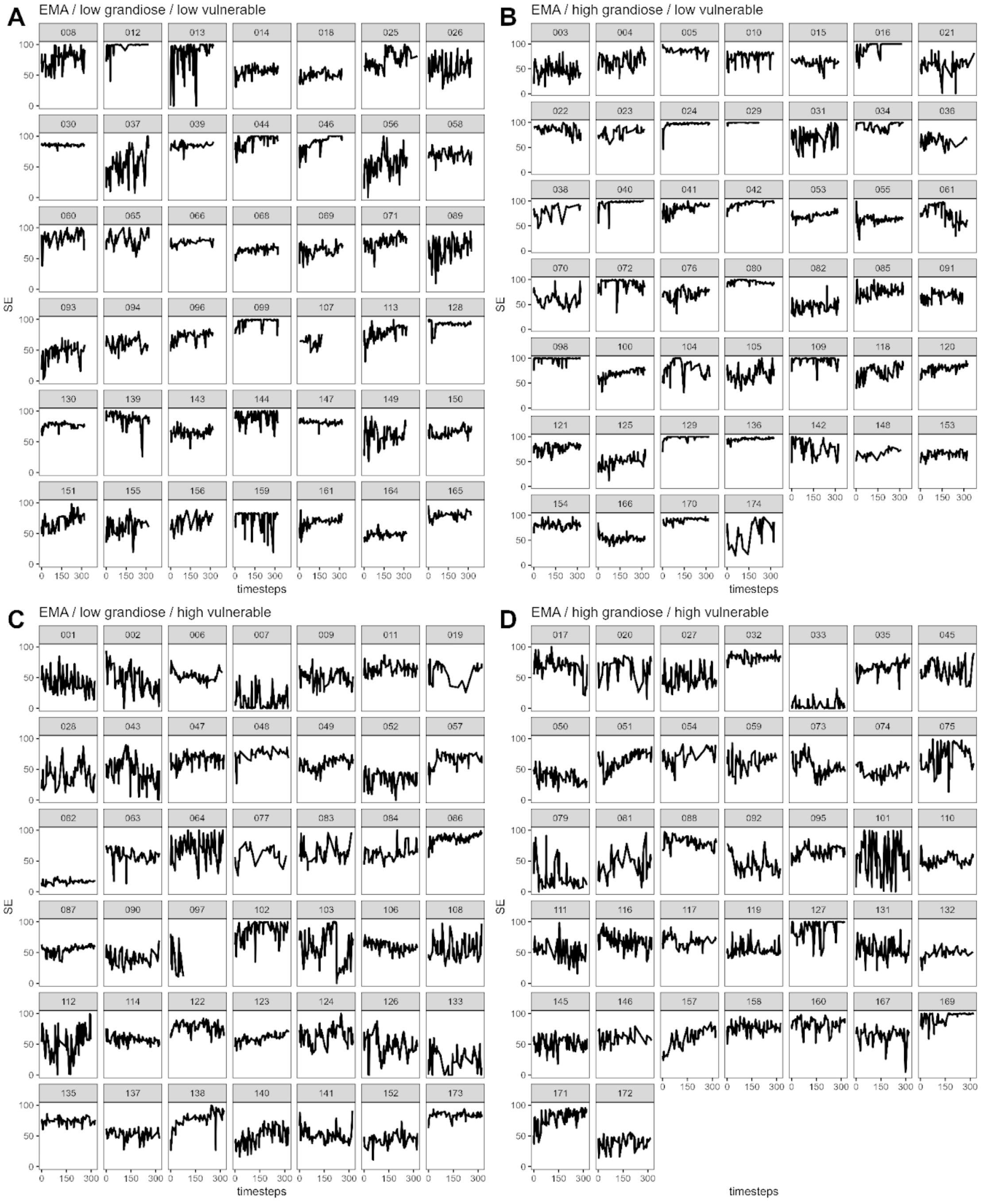

Figure S1. Self-esteem courses of EMA-data subjects for individuals with (A) low grandiose, low vulnerable, (B) high grandiose, low vulnerable, (C) low grandiose, high vulnerable, (D) high grandiose, high vulnerable expressions of trait narcissism measured with the Narcissistic Personality Inventory (NPI; 1) and the Hypersensitive Narcissism Scale (HSNS; 2).

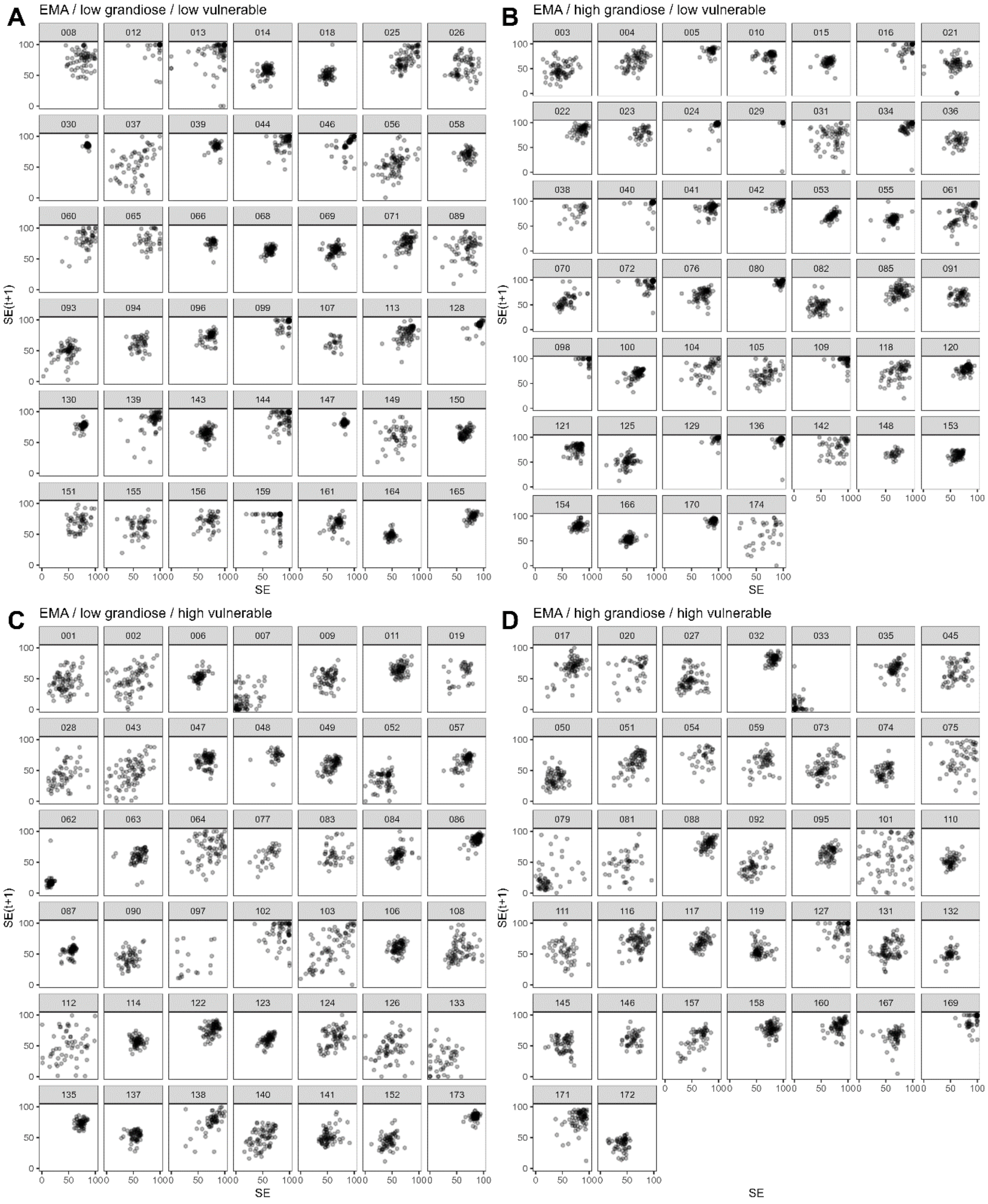

Figure S2. Self-esteem values (for t and t+1) of EMA-data subjects for individuals with (A) low grandiose, low vulnerable, (B) high grandiose, low vulnerable, (c) low grandiose, high vulnerable, (D) high grandiose, high vulnerable expressions of trait narcissism measured with the Narcissistic Personality Inventory (NPI; 1) and the Hypersensitive Narcissism Scale (HSNS; 2).

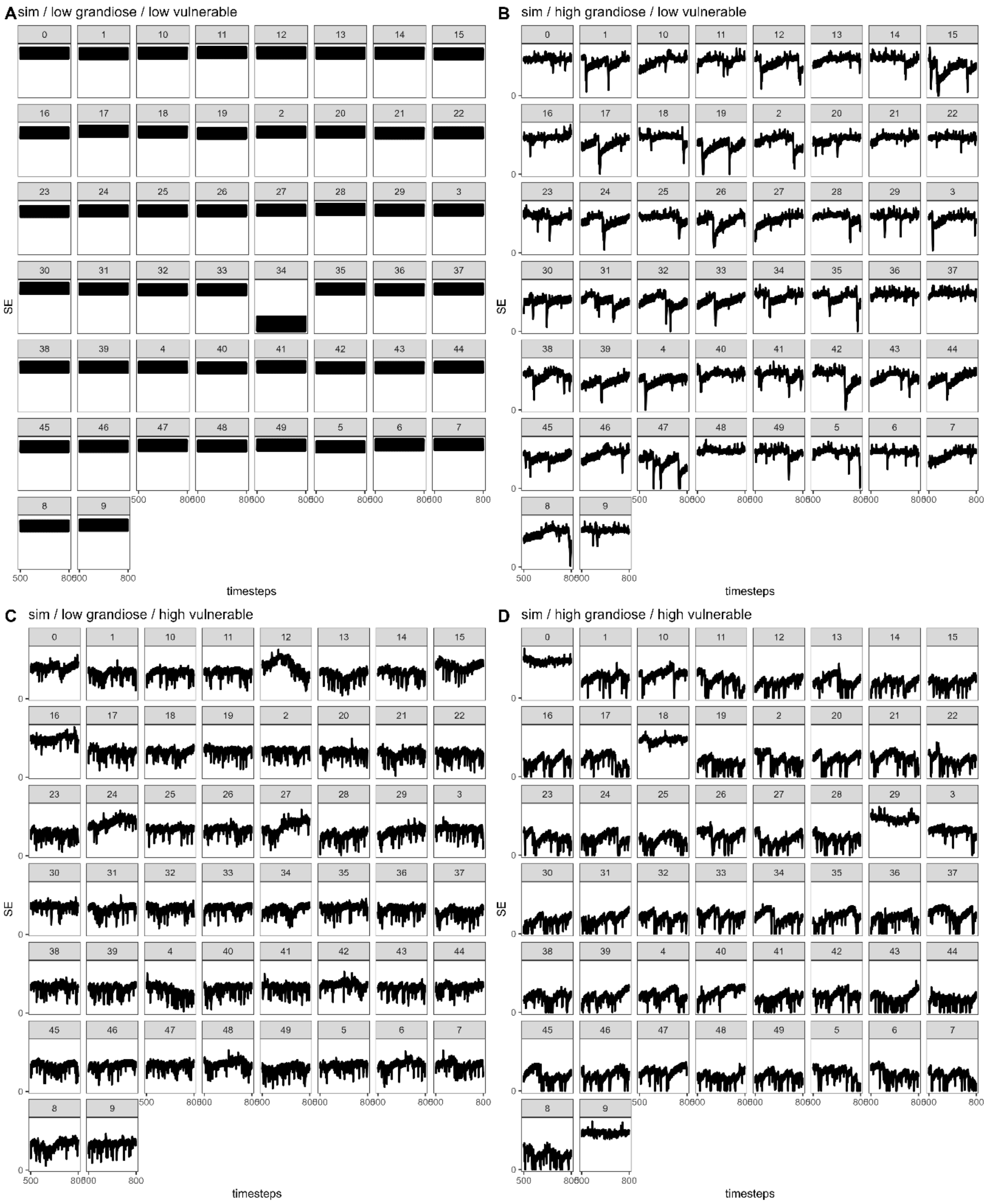

Figure S3. Self-esteem courses of simulated data subjects for agents with (A) low grandiose, low vulnerable, (B) high grandiose, low vulnerable, (c) low grandiose, high vulnerable, (D) high grandiose, high vulnerable expressions of trait narcissism simulated with transformed Five Factor Narcissism Inventory (FFNI; 3) values. FFNI-extraversion represents the threshold to activate admiration, FFNI-antagonism the threshold to activate rivalry.

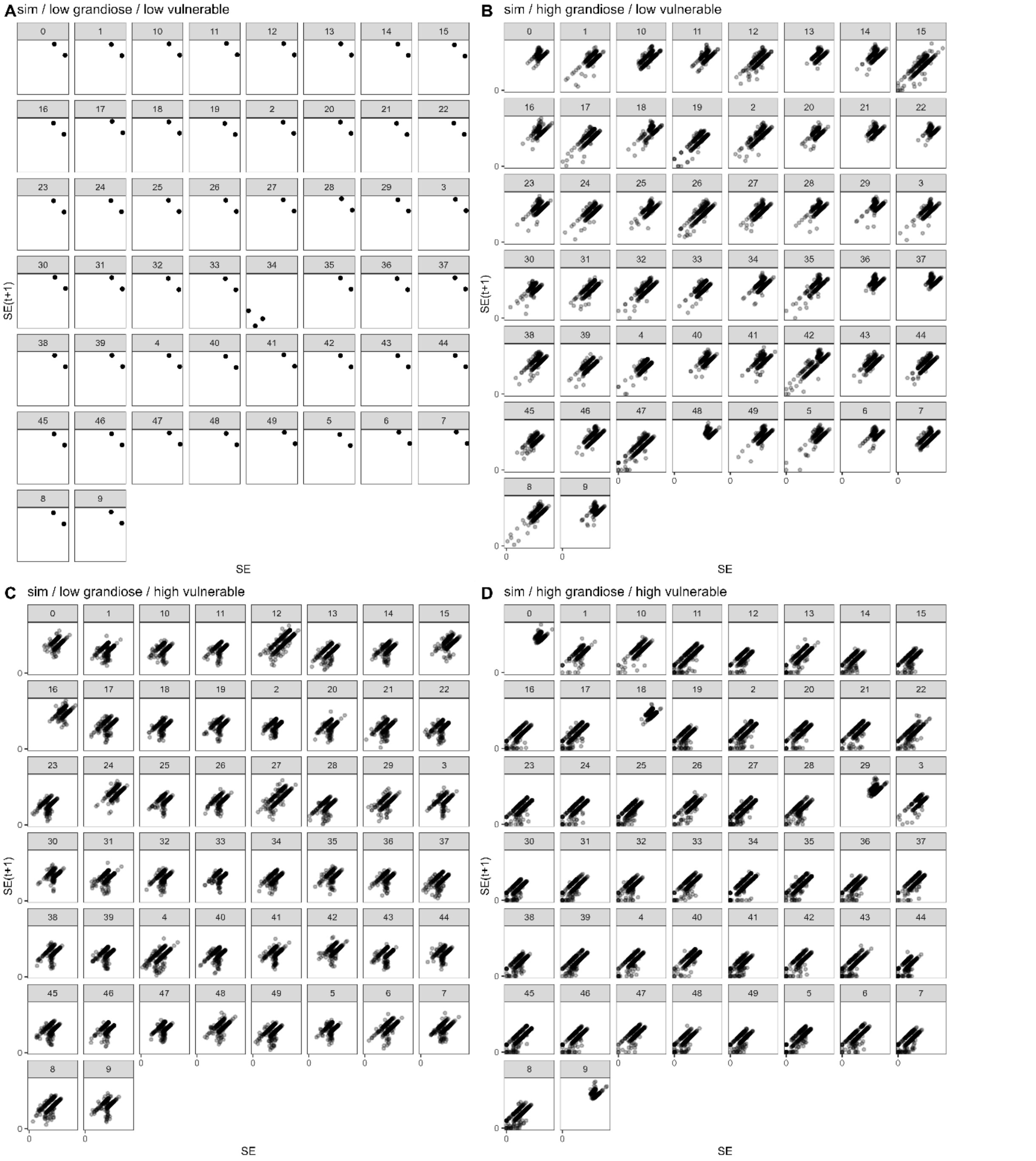

Fig. S4. Self-esteem values (for t and t+1) of simulated data subjects for agents with (A) low grandiose, low vulnerable, (B) high grandiose, low vulnerable, (c) low grandiose, high vulnerable, (D) high grandiose, high vulnerable expressions of trait narcissism simulated with transformed Five Factor Narcissism Inventory (FFNI; 3) values. FFNI-extraversion represents the threshold to activate admiration, FFNI-antagonism the threshold to activate rivalry.

SI B: Supporting Tables

Table S1. Extreme Value Testing SERM.

Para-meter	Self-Esteem					Regulation Behavior[a]			
	Mean	SD	Range	low[b] (%)	high[c] (%)	none (%)	intra (%)	admi (%)	riva (%)
default[d]	0.96	0.12	1.33	8.66	0.03	50.62	48.03	0.22	1.12
TS_{intra}									
0.00125	0.97	0.11	1.20	5.34	0.00	49.95	48.59	0.14	1.33
0.00875	0.95	0.13	1.51	8.63	0.00	51.28	47.38	0.17	1.18
TS_{admi}									
0.005	0.96	0.13	1.31	5.78	0.73	49.10	31.47	17.39	2.04
0.01	0.93	0.17	1.38	11.30	1.11	50.68	31.27	15.28	2.77
0.02	0.91	0.18	1.45	14.75	0.76	50.39	32.60	13.66	3.36
0.14	1.00	0.06	1.01	0.25	0.03	49.55	49.56	0.31	0.57
TS_{riva}									
0.005	0.72	0.19	1.38	56.17	0.22	45.70	32.82	7.90	13.57
0.01	0.73	0.19	1.60	53.70	0.16	46.62	33.37	7.22	12.78
0.0225	0.79	0.18	1.31	35.99	0.08	45.88	36.94	6.59	10.60
0.1575	1.01	0.06	0.49	0.00	0.04	49.89	49.16	0.95	0.00
$rate_{leakage}$									
0.025	1.63	0.31	1.19	11.00	9.50	77.00	23.00	0.00	0.00
0.175	0.58	0.14	1.23	6.84	6.73	45.14	50.67	0.29	3.91
$rate_{tolerance}$									
0.025	0.97	0.13	1.32	3.97	0.04	47.68	48.39	1.06	2.87
0.175	0.99	0.08	1.20	1.58	0.00	49.89	49.38	0.21	0.53
$NFA_{t = 0}$									
0.25	0.66	0.07	0.52	71.00	0.00	68.00	32.00	0.00	0.00
1.75	0.92	0.22	1.65	19.18	6.22	48.08	46.48	0.39	5.05
SE[e] $_{t = 0}$									
0.25	0.86	0.19	1.10	0.25	0.00	53.92	44.59	0.11	1.38
1.75	0.97	0.17	1.75	11.66	3.94	50.50	46.63	0.69	2.17
RSP[f] $_{intra}$									
0.05	0.27	0.13	1.17	97.70	0.00	44.22	50.53	0.08	5.17
0.35	1.12	0.20	1.64	4.79	32.44	57.42	32.81	2.04	7.72

Para-meter	Self-Esteem					Regulation Behavior[a]			
	Mean	SD	Range	low[b] (%)	high[c] (%)	none (%)	intra (%)	admi (%)	riva (%)
RSP[f]$_{admiration|self\_higher}$									
0.0375	0.91	0.18	1.27	17.31	0.03	52.39	45.72	0.21	1.68
0.2625	0.97	0.12	1.38	8.53	0.03	50.62	48.08	0.27	1.03
RSP[f]$_{admiration\ |\ self\_lower}$									
0.05	0.96	0.13	1.19	6.55	0.00	50.40	48.04	0.22	1.34
0.35	0.99	0.09	1.47	2.53	0.08	49.85	48.89	0.26	1.00
RSP[f]$_{rivalry\ |\ self\_higher}$									
0.0125	0.95	0.14	1.23	8.90	0.01	50.91	47.47	0.19	1.44
0.0875	0.97	0.11	1.26	6.64	0.03	50.34	48.35	0.21	1.09
RSP[f]$_{rivalry\ |\ self\_lower}$									
-0.025	0.99	0.8	1.27	1.64	0.01	49.66	49.25	0.25	0.83
-0.175	0.91	0.17	1.18	15.83	0.00	52.34	46.02	0.15	1.49
Varying CA:EA ratio for default values[g]									
49:1	0.97	0.12	1.29	5.30	0.04	50.58	48.13	0.26	1.03
40:10	0.96	0.13	1.29	7.55	0.02	50.76	47.84	0.21	1.19
25:25	0.96	0.13	1.33	6.91	0.02	50.62	48.05	0.17	1.16

Note. For testing the extreme values, the default values were each multiplied by 0.25 (lower value) and 1.75 (higher value). An exception is made for the thresholds for the activation of admiration and rivalry, where two additional small values (0.005 and 0.01) were tested to cover a wider range of values.

[a] none = proportion of no regulation behavior in percent; intra = proportion of intra-individual regulation in percent, admi = proportion of admiration regulation in percent, riva = proportion of rivalry regulation in percent.

[b] low = percentage of self-esteem values lower than 75 % of the initial self-esteem.

[c] high = percentage of self-esteem values higher than 125 % of the initial self-esteem.

[d] TS_{intra} = 0.005, TS_{admi} = 0.08, TS_{riva} = 0.09, $rate_{leakage}$ = 0.1, $rate_{tolerance}$ = 0.1, $NFA_{t=0}$ = 1, $SE_{t=0}$ = 1, RSP_{intra} = 0.2, $RSP_{admiration\ |\ self\_higher}$ ~ N(0.15, 0.1), $RSP_{admiration\ |\ self\_lower}$ ~ N(0.2, 0.1), $RSP_{rivalry\ |\ self\_higher}$ ~ N(0.05, 0.15), $RSP_{rivalry\ |\ self\_lower}$ ~ N(-0.1, 0.15)

[e] self-esteem (SE)

[f] response (RSP) to an agent's self-esteem regulation behavior, depending on the type of regulation behavior (admiration or rivalry) and the comparison of the self-esteem of the regulating agent with the responding agent ($self_{higher}$ indicates that the self-esteem of the regulating agent is higher, $self_{lower}$ indicates that the self-esteem of the responding agent is higher)

[g] Different ratios of control agents (CA; default values) and experimental agents (EA; changed values): 49 CA to 1 EA (default), 40 CA to 10 EA and 25 CA to 25 EA.

Table S2. Overview of SERLE Calibration Results for Contingent Responses to Behavior and Noncontingent Learning Experiences

	TS_{admi}		TS_{riva}	
Parameter	Mean	*SD*	Mean	*SD*
default[a]	0.0552	0.0044	0.1169	0.0077
Devaluing[b]				
1	0.0742	0.0029	0.0066	0.0007
10	0.0577	0.0049	0.0782	0.0072
100	0.0563	0.0040	0.1165	0.0069
Overvaluing[b]				
1	0.0055	0.0000	0.1019	0.0074
10	0.0394	0.0039	0.1163	0.0081
100	0.0576	0.0040	0.1201	0.0079
Overvaluing and Devaluing[c]				
1	0.0055	0.0000	0.0060	0.0005
10	0.0442	0.0044	0.0785	0.0065
10 (40:10)[d]	0.0430	0.0038	0.0809	0.0072
10 (25:25)[d]	0.0483	0.0040	0.0933	0.0077
100	0.0648	0.0049	0.1198	0.0085
Indifference[e]				
0 / 0 / 0 / 0	0.0986	0.0045	0.1026	0.0062
0 / 0 / 0 / -0.1	0.0970	0.0075	0.1276	0.0082
0 / 0 / -0.2 / -0.2	0.0978	0.0069	0.1311	0.0078
0 / 0 / 0.3 / -0.3	0.0970	0.0072	0.1270	0.0082
Praise[e]				
0.3 / 0.3 / 0.3 / 0.3	0.0422	0.0045	0.0550	0.0041
0.6 / 0.6 / 0.6 / 0.6	0.0373	0.0031	0.0531	0.0038
0.3 / 0.5 / 0.5 / 0.3	0.0391	0.0034	0.0538	0.0038

Note. Unless stated otherwise, the values for response and non-contingent feedback match the default condition.
[a] Response values correspond to those listed in SI Appendix 1.A Model Structure. Values for over- and devaluation are 100, i.e. a probability of 1 % for over- or devaluation. All other values are equivalent to the default values named in SI Appendix Table S1
[b] Probability of over-/devaluing an agent in the same cell equals 1 divided by given value.
[c] Similar values for over- and devaluation.
[d] Changed ratio of control agents (no learning implemented) and experimental agents (learning implemented) from 49:1 to 40:10 and 25:25.
[e] Given values refer to the reactions according to the pattern $RSP_{admiration\ |\ self\_higher}$ / $RSP_{admiration\ |\ self\_lower}$ / $RSP_{rivalry\ |\ self\_higher}$ / RSP_{self_lower}.

Table S3. Results of Simulation Study 1.

Para-meter	Self-Esteem					Regulation Behavior[a]			
	mean	SD	range	low[b] (%)	high[c] (%)	none (%)	intra (%)	admi (%)	riva (%)
default[d]	0.96	0.12	1.33	8.66	0.03	50.62	48.03	0.22	1.12
TS_{admi}									
0.0055	0.89	0.17	1.44	10.05	2.48	48.82	31.20	16.78	3.20
0.025	0.95	0.15	1.46	7.59	1.40	48.87	34.06	14.51	2.56
0.05	0.97	0.12	1.34	5.07	0.34	49.91	43.08	5.38	1.63
TS_{riva}									
0.0055	0.76	0.18	1.41	14.76	15.91	45.40	33.01	7.02	14.56
0.025	0.84	0.17	1.45	11.46	5.49	45.56	37.66	6.24	10.53
0.05	0.98	0.10	1.35	3.85	0.08	48.62	47.47	2.13	1.78

Note. In the first simulation study, we examined the impact of varying activation thresholds for admiration and rivalry on self-esteem- and self-esteem regulation-related metrics (e.g., self-esteem mean, self-esteem variance, frequency of admiration).

[a] none = proportion of no regulation behavior in percent; intra = proportion of intra-individual regulation in percent, admi = proportion of admiration regulation in percent, riva = proportion of rivalry regulation in percent.

[b] low = percentage of self-esteem values lower than 75 % of the initial self-esteem.

[c] high = percentage of self-esteem values higher than 125 % of the initial self-esteem.

[d] TS_{intra} = 0.005, TS_{admi} = 0.08, TS_{riva} = 0.09, $rate_{leakage}$ = 0.1, $rate_{tolerance}$ = 0.1, $NFA_{t=0}$ = 1, $SE_{t=0}$ = 1, RSP_{intra} = 0.2, $RSP_{admiration \mid self\_higher}$ ~ N(0.15, 0.1), $RSP_{admiration \mid self\_lower}$ ~ N(0.2, 0.1), $RSP_{rivalry \mid self\_higher}$ ~ N(0.05, 0.15), $RSP_{rivalry \mid self\_lower}$ ~ N(-0.1, 0.15)

Table S4. Conditions of Simulation Study 2.

Condition	Overvaluation and Praise	Devaluation and Indifference	Control Condition
Probability of being overvalued	100 %	1 %	1 %
Probability of being devalued	1 %	100 %	1 %
Response to admiration	positive<br>$RSP_{admiration,\ self\_higher} \sim N(0.30, 0.05)$<br>$RSP_{admiration,\ self\_lower} \sim N(0.50, 0.10)$	none<br>$RSP_{admiration,\ self\_higher} \sim N(0.00, 0.05)$<br>$RSP_{admiration,\ self\_lower} \sim N(0.00, 0.10)$	positive<br>$RSP_{admiration,\ self\_higher} \sim N(0.15, 0.10)$<br>$RSP_{admiration,\ self\_lower} \sim N(0.20, 0.10)$
Response to rivalry	positive<br>$RSP_{rivalry,\ self\_higher} \sim N(0.50, 0.05)$<br>$RSP_{rivalry,\ self\_lower} \sim N(0.30, 0.10)$	none<br>$RSP_{rivalry,\ self\_higher} \sim N(0.00, 0.05)$<br>$RSP_{rivalry,\ self\_lower} \sim N(-0.10, 0.10)$	negative<br>$RSP_{rivalry,\ self\_higher} \sim N(0.05, 0.15)$<br>$RSP_{rivalry,\ self\_lower} \sim N(-0.10, 0.15)$

Note. The three conditions of Simulation Study 2 differ in terms of the levels of non-contingent learning experiences (over- and devaluation) and the response for inter-individual self-esteem regulation (admiration and rivalry). Please note that the probabilities for over- and devaluation represent ideal values, while the actual values are lower as agents do not continuously encounter interaction partners and are therefore not continuously over- or devalued.

Table S5. Results of Simulation Study 2.

Condition	Overvaluation and Praise	Devaluation and Indifference	Control Condition
TS_{admi} (M, SD)	0.0055 (0.00)	0.083 (0.003)	0.056 (0.004)
TS_{riva} (M, SD)	0.0576 (0.0037)	0.007 (0.001)	0.113 (0.007)
$SE_{t=1000}$	2.164	0.586	0.892
$NFA_{t=1000}$	1.978	0.515	0.810
Non-contingent Learning Experiences			
Overvaluation (M, SD)	377.755 (18.118)	3.562 (1.624)	3.96 0 (1.993)
Devaluation (M, SD)	3.729 (1.957)	375.048 (17.227)	3.838 (1.915)
Contingent Learning Experiences			
successful admiration	71.79 (8.201)	1.180 (0.914)	43.570 (5.873)
non-successful admiration	0.120 (0.356)	9.370 (2.977)	10.600 (3.342)
successful rivalry	42.670 (6.655)	1.510 (1.087)	10.760 (3.134)
non-successful rivalry	0.370 (0.597)	36.860 (6.084)	37.910 (5.013)

Note. The values represent the mean and standard deviation of the parameter after 1000 timesteps, averaged over 100 iterations for one experimental agent. Non-contingent learning experiences refer to the external over- or devaluation. A contingent learning experience after regulating self-esteem inter-individually (i.e., with admiration or rivalry) is considered successful if the difference between need for admiration and self-esteem declines below the threshold for activating intra-individual regulation (TS_{intra}).

Table S6. Results of Simulation Study 4.

	LG, LV		HG, LV		LG, HV		HG, HV	
NPI[a]	low		high		low		high	
HSNS[b]	low		low		high		high	
	EMA	SIM	EMA	SIM	EMA	SIM	EMA	SIM
n	42	50	46	50	42	50	37	50
FFNI[d] agency (*SD*)	98.640<br>(*19.077*)		126.413<br>(*21.755*)		94.551<br>(*20.964*)		132.793<br>(*18.906*)	
FFNI[d] antagonism (*SD*)	148.185<br>(*26.913*)		171.226<br>(*29.140*)		171.271<br>(*26.819*)		218.657<br>(*35.255*)	
TS_{admi} (*SD*)	-	0.08<br>(*0.001*)	-	0.02<br>(*0.001*)	-	0.07<br>(*0.001*)	-	0.0055<br>(*0.001*)
TS_{riva} (*SD*)	-	0.09<br>(*0.001*)	-	0.08<br>(*0.001*)	-	0.009<br>(*0.001*)	-	0.008<br>(*0.001*)
rank agency / TS_{admi}[f]	3	3	2	2	4	4	1	1
FFNI-agency / TS_{admi} (z-stand.)[d]	-0.544	1.140	0.509	-0.752	-0.699	0.820	0.751	-1.208
rank antagonism / TS_{rivai}[f]	4	4	3	3	2	2	1	1
FFNI-antagonism / TS_{riva} (z-stand.)[d]	-0.723	1.126	-0.123	0.865	-0.121	-0.983	1.113	-1.008
Self-Esteem (SE) Related Values[e] (z-standardized)								
SE Mean (*SD*)	0.269<br>(*0.612*)	0.886<br>(*0.178*)	0.422<br>(*0.690*)	0.520<br>(*0.193*)	-0.509<br>(*0.753*)	-0.390<br>(*0.219*)	-0.278<br>(*0.818*)	-1.016<br>(*0.575*)
SE Median (*SD*)	0.346<br>(*0.662*)	0.869<br>(*0.235*)	0.501<br>(*0.733*)	0.602<br>(*0.166*)	-0.493<br>(*0.797*)	-0.441<br>(*0.212*)	-0.236<br>(*0.910*)	-1.010<br>(*0.565*)
SE Variance (*SD*)	0.288<br>(*0.259*)	0.061<br>(*0.031*)	0.236<br>(*0.197*)	0.367<br>(*0.123*)	0.415<br>(*0.360*)	0.386<br>(*0.169*)	0.396<br>(*0.335*)	0.502<br>(*0.196*)
SE Range (*SD*)	2.336<br>(*0.999*)	1.996<br>(*1.179*)	2.206<br>(*0.795*)	4.733<br>(*0.538*)	2.616<br>(*0.940*)	4.157<br>(*0.690*)	2.649<br>(*0.801*)	4.151<br>(*0.895*)
SE Extreme High Values[g] (*SD*)	7.74 %<br>(*0.073*)	0.34 %<br>(*0.005*)	7.28 %<br>(*0.072*)	9.32 %<br>(*0.044*)	14.47 %<br>(*0.115*)	10.32 %<br>(*0.029*)	12.97 %<br>(*0.106*)	22.68 %<br>(*0.071*)
SE Extreme Low Values[h] (*SD*)	3.89 %<br>(*0.078*)	0.19 %<br>(*0.006*)	2.86 %<br>(*0.061*)	2.45 %<br>(*0.023*)	12.20 %<br>(*0.138*)	13.04 %<br>(*0.063*)	10.86 %<br>(*0.127*)	22.47 %<br>(*0.073*)

Note.
[a] NPI = Narcissistic Personality Inventory (NPI; (2) trait value.
[b] HSNS = Hypersensitive Narcissism Scale (HSNS; 2) trait value.
[c] EMA = Ecological Momentary Assessment Data, SIM = simulated data.
[d] FFNI-values (sum scores) refer to the EMA data and therefore to a trait characteristic (higher values mean, higher values indicate a stronger expression of the trait. Simulated

values pertain to thresholds for activating a behavior, with lower values representing a stronger expression of a trait/behavioral tendency.

[e] Mean of self-esteem parameters for all single subjects/agents (e.g., SE Mean represents the mean of all subject's/agent's self-esteem mean over time).

[f] The ranking values order the trait expressions by their magnitude, with lower values indicating a stronger expression of the trait. For the FFNI values, ranking 1 corresponds to the highest trait value, and ranking 4 to the lowest. For the threshold values, lower values indicate a high trait expression (a lower threshold corresponds to a higher probability of regulating self-esteem with this behavior). Therefore, the lowest threshold values are ranked 1, and the highest threshold values are ranked 4. The ranks were derived from the FFNI data and applied to the simulation data. Rank 1 always corresponds to the tested threshold minimum (as seen in Study 1), while rank 4 corresponds to the default values (TS_{admi} = 0.08, TS_{riva}= 0.09). Ranks 2 and 3 were determined through systematic testing within the given boundaries and were aligned with the FFNI values.

[g] low = percentage of self-esteem values lower than 75 % of the initial self-esteem.

[h] high = percentage of self-esteem values higher than 125 % of the initial self-esteem.

SI C: Supporting Text Information

1. Model Structure

Our model structure is based on six theoretical assumptions: the Self-Discrepancy Theory (4, 5), the Hierometer Theory (6), the assumption of leaky self-esteem (7, 8), the Narcissism Admiration and Rivalry theory (9, 10), the etiological theories of narcissism-imprinting parenting styles (11–16), and assumptions on basic Reinforcement Learning mechanisms (17–19). We formalized these six assumptions forming a structure of different intertwined control circuits regulating self-esteem of which we present each part in detail in the following.

(A) Self-Esteem Regulation (SER) Model

The SER model builds on the evidence of strong oscillating self-esteem in narcissism (20, 21) and its association to inter-individual regulation behavior (22, 23). It links these two core components to the behavior-mobilizing difference between need for admiration and self-esteem, the responses of other agents to the inter-individual regulation behavior (admiration or rivalry) and the self-esteem leakage. These theoretical assumptions lead to the SER model depicted in Fig. 1. The comprehensive model estimation of self-esteem in each new iteration is depicted in the equation

$$SE_{t+1} = SE_t + RSP_{t(A_t,S_t)} - L_{t(SE_t)}$$

where (SE_{t+1}) represents the self-esteem in the next iteration, (SE_t) the current self-esteem, (RSP_t) the response of other agents to self-esteem regulation behavior, which depends on the employed self-esteem regulation behavior (A_t for action), and the status (S_t) of the agent in a social hierarchy, and the relative self-esteem leakage (L_t) in each iteration. The mathematical formalization of single model components and dynamics are described in the following.

Self-Discrepancy Theory and Initialization of Regulation Behavior

The initiation of regulation behavior is estimated by the equations

$$DIFF_t = SE_t - NfA_t$$

$$NfA_t = NfA_{t-1} + rate_{tolerance} \times DIFF_{t-1}$$

where ($DIFF_t$) represents the difference between the need for admiration (NfA_t) and SE_t of the current and ($DIFF_{t-1}$) of the previous iteration, (NfA_t) the current and (NfA_{t-1}) the previous need for admiration and $rate_{tolerance}$ the set rate to increase or decrease the need for admiration. We emphasize the temporally dynamic association of self-esteem and the need for external approval. We assume that the need for admiration represents a latent expectation of measurable self-esteem. The need for admiration is gradually adjusted to recent experiences, allowing for long-term adaptation to positive or negative environmental feedback (i.e., rate for tolerance development $rate_{tolerance} = 1.1$). Following the self-discrepancy theory (4, 5), the difference between self-esteem and the expected value is perceived as aversive and forces corresponding regulation behavior. Regulation behavior is initialized if the difference exceeds a behavior-activating threshold.

Hierometer Theory and Response to Regulation Behavior

The response (i.e., increase or decrease in self-esteem) to a displayed regulation behavior is estimated by the equation

$$RSP_t(A_t, S_t) \sim N(\mu_{A,S}, \sigma_{A,S}), with$$

$$S_t = \begin{cases} self_{higher}\ with\ SE_{self} \geq SE_{other} \\ self_{lower}\ with\ SE_{self} < SE_{other} \end{cases} and$$

$$A_t = \begin{cases} intra-individual\ with\ TS_{admi} > DIFF_t \geq TS_{intra}\ or\ no\ cellmate\ available \\ admiration\ with\ (TS_{admi} \leq TS_{riva}\ and\ TS_{riva} > DIFF_t \geq TS_{admi}\ and\ cellmate\ available)\ or \\ with\ (TS_{admi} > TS_{riva}\ and\ DIFF_t \geq TS_{admi}\ and\ cellmate\ available) \\ rivalry\ with\ (TS_{riva} < TS_{admi}\ and\ TS_{admi} > DIFF_t \geq TS_{riva}\ and\ cellmate\ available)\ or \\ with\ (TS_{riva} \geq TS_{admi}\ and\ DIFF_t \geq TS_{riva}\ and\ cellmate\ available) \end{cases}$$

where $(\mu_{A,S})$ represents the action (A_t) and status (S_t) dependent mean of a normal distribution and $(\sigma_{A,S})$ the corresponding standard deviation, (TS_{intra}) the threshold to activate intra-individual regulation, (TS_{admi}) the threshold to activate admiration and (TS_{riva}) the threshold to activate rivalry. The exact values to estimate these normal distributions are listed below. We assume that the activated, inter-individual, regulation behavior primarily occurs in two distinct forms: admiration and rivalry. The initialization of these behaviors follows a hierarchical structure, meaning that admiration is initialized first if TS_{admi} is smaller, or rivalry is initialized first if TS_{riva} is smaller. Depending on environmental characteristics, admiration and rivalry are associated with different consequences for an individual's self-esteem. Following the Hierometer Theory (6), we assume that self-esteem in narcissism can be interpreted as a reflection of the individual's subjectively perceived position in a social hierarchy. Hierarchy-higher individuals tend to receive positive feedback from their environment regardless of their behavior toward hierarchy-lower individuals (6, 24, 25). In our model, this is conceptualized by the higher likelihood for successful interactions regarding the self-esteem related consequences (increase vs. decrease) for the agent with higher self-esteem. If no other agents are available or the difference between self-esteem and need for admiration exceeds only the threshold for intra-individual but not inter-individual behavior, self-esteem is regulated intra-individually. With the learning component deactivated, the thresholds to activate regulation behavior remain constant across all iterations.

Normal Distributions for the Response to Regulation Behavior

Following theoretical assumptions, we assumed different probable responses depending on the displayed behavior (A_t) and the status of the agent (S_t). The two characteristics A and S define the mean and standard deviation of a normal distribution. The response is a random value from the normal distribution $N(\mu_{A,S}, \sigma_{A,S})$. Intra-individual regulation is an exception. Sigma is equal to zero, as we wanted to keep the response to intra-individual constant. As there is no interaction with other agents and thus no status comparison, there is no dependency on status S. Accordingly, the response to intra-individual regulation is uniformly distributed.

Admiration represents charming, confident self-presentation (9, 10), leading to our assumption of a high likelihood for positive responses and a likelihood close to zero for negative responses (higher means and lower standard deviation of the normal distribution compared to rivalry). A lower status represents a “lower” (perceived) rank in a social hierarchy, leading to our assumption of a higher likelihood for positive responses to admiration (i.e., higher means of the normally distributed response values) as a positive reaction from a "higher-ranked" person boosts self-esteem more than that from a lower-ranking one (6, 21) higher means of the normal distribution compared to higher status). A higher status represents a “higher” (perceived) rank in a social hierarchy, leading to our assumption of a lower likelihood for positive responses (i.e., lower means of the normally distributed response values), as a positive reaction from a "lower-ranked" person can boost self-esteem less (6, 21).

Rivalry, on the other hand, represents hostile-aggressive devaluation of others (9, 10), leading to our assumption of a high likelihood for negative responses and a moderate likelihood for positive responses (lower means and higher standard deviations of the normal

distributions compared to admiration). A lower status represents a “lower” (perceived) rank in a social hierarchy, leading to our assumption of a lower likelihood for positive responses (i.e., lower means of the normally distributed response values) as there is no social advantage for the status higher interaction partner to respond positively (6, 21, 26). A higher status represents a “higher” (perceived) rank in a social hierarchy, leading to our assumption of a higher likelihood for positive responses (i.e., higher means of the normally distributed response values) as there might be a social advantage for the status lower interaction partner to respond positively (6, 21, 26). Based on these theoretical assumptions, the exact values were determined through systematic trial and error testing.

$$RSP_{t\,|\,intra-individual} = 0.2$$
$$RSP_{t\,|\,admiration,\,self_{higher}} \sim N(0.15, 0.10)$$
$$RSP_{t\,|\,admiration,\,self_{lower}} \sim N(0.20, 0.10)$$
$$RSP_{t\,|\,rivalry,\,self_{higher}} \sim N(0.05, 0.15)$$
$$RSP_{t\,|\,rivalry,\,self_{lower}} \sim N(-0.10, 0.15)$$

Leaky Self-Esteem

The repeated leakage of self-esteem is estimated by the equation

$$L_t = rate_{leak} \times SE_t$$

where (L_t) represents the leakage and $(rate_{leak})$ the set rate for the leakage in each iteration. We assume that narcissistic individuals strive for recurrent validation (7, 8, 10). We modeled this assumption as constantly leaking self-esteem over time, requiring individuals to counter-regulate. Based on the Hierometer Theory (6), self-esteem represents the result of a continuous social comparison. Without social comparison, self-esteem gradually decreases because of the missing social update of an individual’s own value. As the social environment may change quickly, self-esteem needs to be readjusted steadily to keep it a valid indicator of the social position. The individual’s evaluation must be validated recurrently to prevent a drop in self-esteem (24, 27). In our model, this is conceptualized by the continuous reduction of self-esteem by 10% in each iteration ($rate_{leak}$ = 0.1).

(B) Learning Component (LSER Model)

The learning component builds on assumptions on the learning of behavioral predispositions for self-esteem regulation behavior through the adjustment of behavior-initiation thresholds for admiration and rivalry to environmental feedback. We focus on two learning processes, namely non-contingent narcissism-imprinting parenting styles (over- vs. devaluation) as external influences (non-contingent learning) and contingent basic reinforcement learning mechanisms controlling for the responses to the shown self-esteem regulation behavior (contingent learning). These theoretical assumptions lead to the gray shaded extension of the SER model depicted in Fig. 1. The comprehensive estimation of the learning experiences in each iteration are formalized in the equations

$$TS_{A\,|\,t+1} = TS_{A\,|\,t} - NCL_t - CL_t$$

where (TS_A) represents the threshold to activate (A) inter-individual regulation behavior (admiration or rivalry), (NCL_t) the non-contingent learning experience and (CL_t) the contingent learning experience. The current threshold and both learning experiences are labeled with “t” and form the threshold at time t+1 (TS_{t+1}).

Considering the etiological theories on narcissism, overvaluation and praise on the one and devaluation and indifference on the other hand especially as non-contingent influence were identified as meaningful context parameters, i.e. parental attributes. Therefore, the purpose of the “learning-model” was to explore the impact of contingency and valence of the environmental feedback on learning self-esteem regulation strategies. Contingency is implemented as behavior-related feedback agents receive. In non-contingent conditions, agents also receive feedback without showing any behavior, thus contingency can be understood as the “quantity” of feedback. In contrast, valence can be understood as the qualitative component of the feedback, namely positive (self-esteem increases) or negative (self-esteem decreases or does not increase enough to satisfy the need for self-esteem regulation). Both learning experiences have a specific learning rate (LR = 0.001) and a discount factor which is estimated by the equation

$$DISC_{LE\,|\,t} = \sum_{t'=t-1}^{t} CLE_{t'} \; with$$

$$CLE_{t'} = \begin{cases} 1 \; with \; (SC_{t'} = SC_{t-1} \; and \; A_{t'} = A_{t-1}) or \; NCL_{t'} = \; NCL_{t-1} \\ 0 \; with \; SC_{t'} \neq SC_{t-1} \; or \; A_{t'} \neq A_{t-1} \; or \; NCL_{t'} \neq NCL_{t-1} \end{cases}$$

where ($DISC_{LE\,|\,t}$) represents the discount factor of a learning experience (LE) (i.e., defined by regulation behavior and success or the type of non-contingent feedback), ($CLE_{t'}$) the current learning experience, (SC) the success of a behavior (i.e., difference between self-esteem and need for admiration of the current iteration is lower than TS_{intra}), (A) the regulation behavior, and (NCL) the non-contingent learning experience (i.e., over- or devaluation). CLE is defined by either the regulation behavior and the success of the behavior or the non-contingent external feedback (i.e., over- or devaluation). The discount factor determines how many of the last ten learning experiences correspond to CLE. Following the assumptions of frequency-based learning, the discount factor is higher and the effect for learning threshold values is lower the more often an agent experiences similar learning experiences. The influence of the current learning experience on threshold learning is lower the more frequent the same learning experience occurred in past iterations.

Non-Contingent Learning: Etiological Theories on Narcissism-Imprinting Parenting Styles

The non-contingent learning is estimated by the equation

$$NCL_t = TS_{A\,|\,t} \times LR \times DISC_{LE\,|\,t}$$

where (NCL_t) represents the non-contingent learning, ($TS_{A\,|\,t}$) the threshold for activating (A) inter-individual regulation behavior, (LR) the learning rate, and ($DISC_{LE\,|\,t}$) the discount rate depending on the (LE) learning experience. In conditions with non-contingent feedback, agents repeatedly receive external over- or devaluation. Overvaluation reduces the threshold for activating admiration behavior, i.e., increases the probability to regulate self-esteem with admiration. Conversely, the threshold for activating rivalry is reduced through devaluation, i.e., the probability to regulate self-esteem with rivalry increases.

Contingent Learning: Reinforcement Learning Mechanisms

The contingent learning is estimated by the equation

$$CL_t = \; TS_{A\,|\,t} \times SC_t \times LR \times DISC_{LE\,|\,t}, with$$

$$SC_t = \begin{cases} 1\ [no] with\ DIFF_t \leq\ TS_{intra} \\ -1\ [yes] with\ DIFF_t >\ TS_{intra} \end{cases}$$

where (CL_t) represents the contingent learning, ($TS_{A\,|\,t}$) the threshold for activating (A) inter-individual regulation behavior, (SC_t) the success of a behavior, (LR) the learning rate, and ($DISC_{LE\,|\,t}$) the discount rate depending on the (LE) learning experience. Agents track if a regulation strategy succeeds (i.e., self-esteem increases enough to satisfy the regulation need) or not (i.e., need to regulate remains). The learning component follows basic reinforcement learning mechanisms: success or reward increases the probability to use a regulation strategy again, failure decreases it. The characteristics of the environment (in terms of contingency and valence) determine the likelihood for reward or failure. Behavior in an overvaluing and praising environment is mostly appreciated and reinforced while the opposite is the case in devaluing contexts (11, 28).

2. Calibration and Validation of the Model

The initial stage of model development involved constructing the intra-individual regulation circuit for individual agents, which was subsequently expanded to incorporate interactional elements. Calibration of the model proceeded iteratively during the model construction phase, employing a "trial and error" approach. Upon introducing a new component, potential parameter values were systematically tested and visually assessed for plausibility concerning expected self-esteem patterns. This process identified value ranges for each variable that induced consistent self-esteem oscillations(equilibrium). This approach also serves as an assessment of the face validity of the model, which was continuously evaluated and verified throughout the process.

The model syntax was continuously checked during the entire modeling process to debug the code. Using print statements, the code functionality was tested again and again, especially when new model components were implemented. Stress tests with extreme values and values that should undermine the model function were also carried out during the process. Also, the debugger implemented in PyCharm was used. With increasing complexity, visual checks of the model as well as spot tests became more and more important, especially for monitoring the self-esteem dynamic. For this purpose, a visualization-tool included in the Mesa framework to animate the model in "real time" was used. This enabled a step-by-step tracking of the behavior of individual agents while monitoring specific parameters like self-esteem. The plausibility of the model was continuously discussed with a model-familiar working group and double-checked with a verification-checklist according to Grimm and colleagues (29).

Subsequent to establishing default values, a systematic model test was conducted using extreme parameterizations. At this stage, learning parameters were not included. To cover a broad range of values, tests were conducted at 25% and 175% of each parameter, with 50 runs of 1000 steps each. Descriptive parameters of self-esteem (median, range, standard deviation, extreme values) and the frequency of regulation behaviors were collected on a run-by-run basis for both Control Agents (CAs) and Experimental Agents (EAs). Additionally, self-esteem trajectories were plotted for individual agents and averaged across all runs and groups to elucidate self-esteem behavior. Further exploration involved testing different ratios of CAs to EAs within specific parameter ranges (e.g., 40:10 ratio and 25:25 ratio for CA:EA). As no significant differences were observed in the output values, a ratio of 49:1 for CAs to EAs was adopted. We set the temporal resolution for subsequent model investigations to 200 timesteps, as no discernible "new patterns" emerged beyond this period. A comprehensive summary of all parameterizations and corresponding outputs from model runs conducted for final calibration is provided in see SI Appendix Table S1.

Based on the default values of the SERM, we expanded the learning component by incorporating the learning mechanisms of EAs, non-contingent feedback, and discount values for learning experiences. The rationale behind calibrating the learning environment was as follows: In a "control environment" where displayed behavior is rewarded contingently and there are no external influences, the resulting regulation behavior should not lead to irregular oscillations of self-esteem, thus threshold values should remain close to the default. In contrast, in strongly over- or devaluing environments with non-contingent feedback, altered threshold values for admiration and rivalry should be observed (see SI Appendix Table S2).

We tested various combinations of the reaction and non-contingent feedback conditions each over 1000 steps in 50 runs (for detailed results, see Table SI.1). As no differences emerged for various CA:EA ratios we retained the 49 CA to 41 EA ratio. Following an observation of a distinct learning trend emerging after approximately 800 to 900 timesteps, we defined 1000 timesteps as the temporal resolution of the model. We also tested two additional ratios of CAs to EAs (40:10 and 25:25) for congruent and contingent, overvaluing-praising, and devaluing-indifferent environments. No significant differences in the target values were observed, thus the ratio of CAs to EAs was set to 49:1. All SERM simulations for both activated and deactivated learning component were conducted in a 10x10 grid, with 50 agents randomly placed within it. For batch runs, each simulation was repeated 100 times.

3. Study 4: Replication Real World Data with Simulated Data

To align Five Factor Narcissism Inventory (FFNI; 3) and admiration and rivalry threshold-values, we initially calculated z-standardized values for the means of FFNI antagonism and FFNI agency across all four groups in the EMA data. We ranked the groups by these values and defined the model values for each group based on calibration results according to these rankings (i.e., lowest calibrated threshold value for the highest trait expression and so on). These values were then used as threshold-means (of a normal distribution with *SD* = 0.01) for simulating the data in each group.

Each simulation involved 50 agents per group, each assigned a value from the corresponding normal distribution for the thresholds of admiration and rivalry. As results of the simulation, we obtained the individual threshold for admiration and rivalry, as well as the trajectory of self-esteem over time (1000 timesteps) for each agent. For further analysis, we only utilized 300 timesteps to make the simulated data more comparable to the EMA data, which were collected over 300 time units (hours). All metrics (thresholds and self-esteem) were z-standardized. Additionally, we inverted the thresholds to make them directly comparable (higher value equals more pronounced trait) to the FFNI traits of extraversion and antagonism. The trait and threshold values for both datasets (EMA and SIM) can be found in SI Appendix Table S6 as well as several self-esteem parameters.

Appendix References

1. R. N. Raskin, C. S. Hall, A Narcissistic Personality Inventory. *Psychol. Rep.* **45**, 590–590 (1979).

2. H. M. Hendin, J. M. Cheek, Assessing Hypersensitive Narcissism: A Reexamination of Murray's Narcism Scale. *J. Res. Personal.* **31**, 588–599 (1997).

3. E. Jauk, G. Olaru, E. Schürch, M. D. Back, C. C. Morf, Validation of the German Five-Factor Narcissism Inventory and Construction of a Brief Form Using Ant Colony Optimization. *Assessment* **30**, 969–997 (2023).

4. M. D. Barnett, P. M. Womack, Fearing, not loving, the reflection: Narcissism, self-esteem, and self-discrepancy theory. *Personal. Individ. Differ.* **74**, 280–284 (2015).

5. X. Xu, E. S. Huebner, L. Tian, Profiles of narcissism and self-esteem associated with comprehensive mental health in adolescents. *J. Adolesc.* **80**, 275–287 (2020).

6. N. Mahadevan, A. P. Gregg, C. Sedikides, Is self-regard a sociometer or a hierometer? Self-esteem tracks status and inclusion, narcissism tracks status. *J. Pers. Soc. Psychol.* **116**, 444–466 (2019).

7. R. Baumeister, K. D. Vohs, Narcissism as addiction to esteem. *Psychol. Inq.* **12**, 206–210 (2001).

8. C. C. Morf, F. Rhodewalt, Unraveling the Paradoxes of Narcissism: A Dynamic Self-Regulatory Processing Model. *Psychol. Inq.* **12**, 177–196 (2001).

9. M. Back, *et al.*, Narcissistic Admiration and Rivalry: Disentangling the Bright and Dark Sides of Narcissism. *J. Pers. Soc. Psychol.* **105**, 1013–1037 (2013).

10. M. D. Back, "The Narcissistic Admiration and Rivalry Concept" in *Handbook of Trait Narcissism: Key Advances, Research Methods, and Controversies*, A. D. Hermann, A. B. Brunell, J. D. Foster, Eds. (Springer International Publishing, 2018), pp. 57–67.

11. E. Brummelman, *et al.*, Origins of narcissism in children. *Proc. Natl. Acad. Sci.* **112**, 3659–3662 (2015).

12. O. F. Kernberg, *Borderline Conditions and Pathological Narcissism* (Rowman & Littlefield, 1985).

13. O. F. Kernberg, Hass, Wut, Gewalt und Narzissmus. (2016). Available at: https://elibrary.kohlhammer.de/book/10.17433/978-3-17-029724-1 [Accessed 27 June 2024].

14. H. Kohut, Forms and Transformations of Narcissism. *J. Am. Psychoanal. Assoc.* **14**, 243–272 (1966).

15. H. Kohut, *The restoration of the self* (University of Chicago Press, 1977).

16. T. Millon, G. S. Everly, T. Millon, *Personality and its disorders a biosocial learning approach* (Wiley, 1985).

17. P. Dayan, Y. Niv, Reinforcement learning: The Good, The Bad and The Ugly. *Curr. Opin. Neurobiol.* **18**, 185–196 (2008).

18. R. F. Thompson, W. A. Spencer, Habituation: A model phenomenon for the study of neuronal substrates of behavior. *Psychol. Rev.* **73**, 16–43 (1966).

19. E. L. Thorndike, The Law of Effect. *Am. J. Psychol.* **39**, 212–222 (1927).

20. V. Zeigler-Hill, A. Besser, Full article: A Glimpse Behind the Mask: Facets of Narcissism and Feelings of Self-Worth. (2013). Available at: https://www.tandfonline.com/doi/full/10.1080/00223891.2012.717150 [Accessed 4 July 2024].

21. V. Zeigler-Hill, J. Vonk, Narcissism and Self-Esteem Revisited : The Mediating Roles of Perceived Status and Inclusion. *Identity* **23**, 4–17 (2022).

22. V. L. Freund, G. V. Eisele, F. Peeters, J. Lobbestael, Ripples in the water: Fluctuations of narcissistic states in daily life. *Personal. Disord. Theory Res. Treat.* **15**, 193–206 (2024).

23. K. Geukes, *et al.*, Puffed-up but shaky selves: State self-esteem level and variability in narcissists. *J. Pers. Soc. Psychol.* **112**, 769–786 (2017).

24. M. R. Leary, Making Sense of Self-Esteem. *Curr. Dir. Psychol. Sci.* **8**, 32–35 (1999).

25. N. Mahadevan, A. P. Gregg, C. Sedikides, W. G. de Waal-Andrews, Winners, Losers, Insiders, and Outsiders: Comparing Hierometer and Sociometer Theories of Self-Regard. *Front. Psychol.* **7** (2016).

26. S. Grapsas, E. Brummelman, M. D. Back, J. J. A. Denissen, The “Why” and “How” of Narcissism: A Process Model of Narcissistic Status Pursuit. *Perspect. Psychol. Sci.* **15**, 150–172 (2020).

27. A. Tesser, N. Crepaz, J. C. Collins, D. Cornell, S. R. H. Beach, Confluence of Self-Esteem Regulation Mechanisms: On Integrating the Self-Zoo. *Pers. Soc. Psychol. Bull.* **26**, 1476–1489 (2000).

28. L. Imbesi, The making of a narcissist. *Clin. Soc. Work J.* **27**, 41–54 (1999).

29. V. Grimm, A. S. A. Johnston, H.-H. Thulke, V. E. Forbes, P. Thorbek, Three questions to ask before using model outputs for decision support. *Nat. Commun.* **11**, 4959 (2020).
